# A Comprehensive View on the Human Antibody Repertoire Against *Staphylococcus aureus* Antigens in the General Population

**DOI:** 10.3389/fimmu.2021.651619

**Published:** 2021-03-10

**Authors:** Tanja C. Meyer, Stephan Michalik, Silva Holtfreter, Stefan Weiss, Nele Friedrich, Henry Völzke, Thomas Kocher, Christian Kohler, Frank Schmidt, Barbara M. Bröker, Uwe Völker

**Affiliations:** ^1^Department Functional Genomics, Interfaculty Institute for Genetics and Functional Genomics, University Medicine Greifswald, Greifswald, Germany; ^2^Department of Immunology, Institute of Immunology and Transfusion Medicine, University Medicine Greifswald, Greifswald, Germany; ^3^Institute of Clinical Chemistry and Laboratory Medicine, University Medicine Greifswald, Greifswald, Germany; ^4^Institute for Community Medicine, University Medicine Greifswald, Greifswald, Germany; ^5^Unit of Periodontology, University Medicine Greifswald, Greifswald, Germany; ^6^Friedrich Loeffler Institute of Medical Microbiology, University Medicine Greifswald, Greifswald, Germany; ^7^Proteomics Core, Weill Cornell Medicine-Qatar, Qatar Foundation-Education City, Doha, Qatar

**Keywords:** *S. aureus*, host-pathogen-interaction, antibody repertoire, anti-*S. aureus* IgG response, anti-*S. aureus* IgA response, immunoproteomics

## Abstract

Our goal was to provide a comprehensive overview of the antibody response to *Staphylococcus aureus* antigens in the general population as a basis for defining disease-specific profiles and diagnostic signatures. We tested the specific IgG and IgA responses to 79 staphylococcal antigens in 996 individuals from the population-based Study of Health in Pomerania. Using a dilution-based multiplex suspension array, we extended the dynamic range of specific antibody detection to seven orders of magnitude, allowing the precise quantification of high and low abundant antibody specificities in the same sample. The observed IgG and IgA antibody responses were highly heterogeneous with differences between individuals as well as between bacterial antigens that spanned several orders of magnitude. Some antigens elicited significantly more IgG than IgA and vice versa. We confirmed a strong influence of colonization on the antibody response and quantified the influence of sex, smoking, age, body mass index, and serum glucose on anti-staphylococcal IgG and IgA. However, all host parameters tested explain only a small part of the extensive variability in individual response to the different antigens of *S. aureus*.

## Introduction

The interactions between *Staphylococcus aureus* and humans span a broad range from unnoticed colonization to severe damage in various diseases including blood-stream infections (1) and recurrent episodes of skin and soft tissue infections (2). Due to the wide spread of *S. aureus* within the population, the resulting high frequency of infections, and an increase of community-acquired antibiotic-resistant strains, the burden on health-care systems has been continuously growing over the past decades (3). Since about 20% of the human population carries staphylococci continuously and the remainder intermittently with transient phases of colonization, a mean carriage rate of 37.2% has been estimated (4, 5). Some subgroups of the population show higher colonization rates, for instance 73–94% in atopic eczema patients (6, 7), 56.4% in insulin-dependent diabetics, and 51.5% in hemodialysis patients (4).

Besides disease-associated variation in carriage rates, multiple host factors have been described that influence the individual chance for a persistent colonization. Apart from genetic host factors [for overview see (8, 9)], additional factors like age, sex, nutritional status, and further lifestyle choices have an impact on the rate of contacts with *S. aureus* and carriage rates. Carriage is highest in children within the first 8 weeks after birth reaching 40–50% in infants and decreases with increasing age (10–12). A sex bias was reported hinting to higher colonization rates in males using culture-based detection methods (12), which might be due to overall higher bacterial loads in males (13). In a large colonization prevalence study in the US, obesity was linked to increased colonization rates (11), and in the Rotterdam Study diabetes and elevated fasting serum glucose showed a correlation to higher colonization rates (14). Active smoking was described to result in lower carriage rates (14), and a combined influence of vitamin D levels in serum and smoking was shown in the Tromsø Staph and Skin Study (15).

The majority of the persistently colonized individuals carry the same strain over long time spans, but also abrupt changes and periods of co-carriage with two different strains were described in a weekly swab study of healthy students conducted by Ritchie et al. (16). A rapid turnover of strains was reported for intermittent carriers with a median carriage duration of 4 weeks and an estimated six episodes within 1 year (16). Applying a staged protocol, the detection of multiple-strain colonization has been improved, detecting up to four different *spa* types in a single nasal swab (17).

It is known that persistently colonized individuals have a higher risk of *S. aureus* bacteremia compared to non-colonized patients when undergoing clinical surgery. This is usually caused by their own colonizing strain (18, 19). Notably, in case of blood stream infection, *S. aureus* carriers have a significantly better chance of survival of severe infections (20). We previously proposed that a pre-established memory immune response to the colonizing strain due to long term exposure and repeated minor infections may confer some degree of clinical protection (21). Indeed, *S. aureus* carriers mount a strain-specific antibody response against their colonizing strain (22). Moreover, high antibody titers at the onset of *S. aureus* blood stream infection are correlated with a milder disease course (23–25).

Depending on the mode of interaction between *S. aureus* and the human host, specific antibody classes are triggered. Since *S. aureus* colonizes and infects mucosal surfaces in humans, the appearance of an anti-staphylococcal IgA response can be expected. IgA occurs in two variants in the human body: the monomeric IgA circulating in the blood stream, and a dimeric form known as secretory IgA which mainly prevents mucosa-associated bacteria from entering the human body (26). IgA has neutralizing properties but comparably low inflammatory potential (27). A strong clonal relationship between serum and gut IgA was shown, allowing the use of serum monomeric IgA as a proxy for the hardly accessible secretory dimeric IgA of mucosae (28). In contrast, an anti-staphylococcal IgG response is likely mounted after invasive episodes. Apart from neutralizing bacterial factors, these antibodies promote inflammation and bacterial clearance by professional phagocytes. Both IgA and IgG with the potential of binding to *S. aureus* have been found in the epidermis showing the importance of this first line of defense (29). Systemic anti-staphylococcal IgG and IgA show an early onset in childhood, with no correlation between the two antibody classes, and not limited to individuals with clinical symptoms of infection (30).

To lower the clinical burden on the human population, various anti-*S. aureus* strategies including antibiosis and vaccination have been tested, but with limited success so far. Multiple strategies to develop a vaccine have failed until now, including active as well as passive immunization approaches (31, 32). The failure of early trials targeting adhesins and surface glyocopolymers forced *S. aureus* researchers to revisit *S. aureus* pathogenesis and potential correlates of protection. Targeting single adhesion factors can fail due to multiple functionally redundant proteins present in *S. aureus*. The focus on opsonophagocytosis may not be as effective as in other pathogens, since many additional virulence factors, including toxins and immune evasion proteins, are involved in *S. aureus* pathogenesis (33). As a consequence, current approaches shifted to the use of multiple targets, e.g., the pore-forming toxins targeted by a monoclonal antibody (34). The selection of protective vaccine candidates targeting the majority of staphylococcal strains is not an easy task due to the high variability of genomes between various strains. Bosi et al. (35) described the variable pangenome to harbor four times as many entries as the core genome common to all strain (35). A detailed understanding of the anti-staphylococcal antibody repertoire in healthy individuals and disease cohorts is essential to identify suitable vaccine targets for future trials.

The anti-staphylococcal antibody repertoire in humans has already been addressed in earlier studies. In a study by Verkaik et al. (36) profiling 19 antigens, it was shown that all included individuals had specific IgG to at least CHIPS and SCIN, underlining the wide spread of *S. aureus* in the human population (36). The authors reported a high inter-individual variability in antibody titers, but also a significant difference between persistent carriers and non-carriers in the IgG and IgA response to toxic shock syndrome toxin (TSST-1) and staphylococcal enterotoxin A, and the IgA response to clumping factor A and B. In patients suffering from acute infection caused by *S. aureus*, Dryla et al. (30) reported increased IgG titers directed against six of 19 staphylococcal antigens (30). Rigat et al. (37) confirmed the “nearly universal” natural exposure of humans to *S. aureus* using a panel of 134 antigens, but found only little changes of the antibody levels due to a clinically significant *S. aureus* infection (37). All the aforementioned studies were limited in their dynamic range as only one serum dilution was analyzed per sample, rendering them prone to saturation and limit-of-detection effects. Additionally, most studies were limited in sample size, with only small numbers of healthy control individuals available for comparison to a disease group.

To overcome these limitations, we applied a high-dynamic-range bead-based multiplex assay (based on the Luminex® system) to 996 individuals of the population-based cohort SHIP-TREND-0. By measuring each sample at seven dilutions, we broadened the detection range of this assay format enabling exact calculation of antibody response values for both low and high titer sera. With our panel of 79 antigens, we describe the extreme heterogeneity of the antibody response in healthy adults and identify multiple host factors that influence the anti-staphylococcal antibody repertoire. Additionally, we shed light on the natural immunogenicity of the included antigens which could guide the selection of vaccine candidates.

## Materials and Methods

### Staphylococcal Antigens

The heterologous expression of most staphylococcal antigens was accomplished using *Escherichia coli* SCS1 cells equipped with a two-plasmid system purchased from Protagen AG (Germany). Sequences were derived from the *S. aureus* strain NCTC 8325 if not indicated otherwise. Five proteins were not suitable for expression in full length and were thus expressed as two separate domains (see Supplementary Table 1 for detailed information). A two-plasmid setup was used for all expressions. The helper plasmid pSE111 was selected via a kanamycin resistance cassette and contained genes coding for the Lac repressor protein and a rare tRNA for arginine. The expression plasmid pQE30NST was selected via an ampicillin resistance cassette and contained the sequence for the desired staphylococcal antigen joined to a N-terminal hexa-His-tag for purification via a multi-cloning site. The expression was controlled by the Lac repressor, allowing an induction using Isopropyl-β-D-1-thiogalactopyranoside (IPTG). The strains were cultivated in super broth supplemented with 100 μg/mL ampicillin and 30 μg/mL kanamycin at 37°C with orbital shaking at 250 rpm starting with an overnight pre-culture set-up as a dilution series followed by the main cultivation inoculated to an optical density at 540 nm (OD_540nm_) of 0.1. When the culture reached an OD_540nm_ of 2.0, IPTG was added to a concentration of 1 mM. Two hours after induction, the cells were harvested by centrifugation. The bacterial pellet was washed with PBS, then flash frozen in liquid nitrogen, and stored at −80°C until cell disruption.

The expression of truncated, non-functional Protein A (Spa_trunc) was accomplished using *E. coli* BL21 (DE3) pLysS_SAC_spa_His, with the *spa* cassette inserted into pASK-IBA33+ (IBA GmbH, Germany) under the control of a tetracycline-inducible promoter with an ampicillin resistance cassette for selection. Cultivation was performed in super broth supplemented with 100 μg/mL ampicillin and 25 μg/mL chloramphenicol. The main culture was inoculated with growing bacteria from an overnight pre-culture to an OD_540nm_ of 0.1; target protein expression was induced at an OD_540nm_ of 0.5 by adding anhydrotetracycline to a concentration of 0.2 μg/mL. The cells were harvested 2 h after induction by centrifugation, washed with PBS, flash frozen in liquid nitrogen, and stored at −80°C.

Cell disruption was performed using glass beads for mechanical disruption within a FastPrep cell homogenizer (Thermo Savant, US). The harvested pellets were resuspended in lysis/equilibration/binding buffer and then shaken with the glass beads three times for 30 s with cooling on ice in between. After centrifugation of the glass beads the supernatant was collected. For purification of the His-tagged proteins Protino® 96 Ni-IDA purification plates were used according to the manufacturer's protocol (MACHEREY-NAGEL GmbH & Co. KG, Germany). The purity of the protein products was verified by one-dimensional sodium dodecyl sulfate polyacrylamide gel electrophoresis followed by Coomassie staining and additionally by mass spectrometric analyses of the eluates.

### Human Plasma Samples From SHIP-TREND-0 Cohort

Human plasma samples were derived from the SHIP-TREND-0 cohort of the Study of Health in Pomerania (SHIP). In brief, a stratified random sample of adults living in the region of Western Pomerania was invited for a personal interview as well as medical examinations to assess their state of health. A full list of initial instruments of investigation can be found in Völzke et al. (38), followed by multiple subsequent initiatives like the description of the molecular epidemiology of nasal *S. aureus* isolates obtained from this cohort by Holtfreter et al. (39). A subset of 996 subjects was used for this study, for detailed characteristics see Table 1. The study protocol of the SHIP-TREND-0 cohort was approved by the local ethics committee of the University of Greifswald (registration no. BB39/08) with all participants giving informed written consent.

**Table 1:** Characteristics of the study cohort.

	**Carrier**	**Non-carrier**	**No information**
Total	251 (25.5%)	733 (73.6%)	12 (1.2%)
Men	118 (47.0%)	319 (43.5%)	3 (25.0%)
Mean age	49.0	50.5	46.5
Current smoking	57 (22.7%)	158 (21.6%)	4 (33.3%)
Never smoked	110 (43.8%)	305 (41.6%)	4 (33.3%)
Reported allergy	76 (30.3%)	221 (30.2%)	4 (33.3%)
Mean BMI	26.9	27.5	27.3
Mean serum glucose [mmol/L]	5.39	5.37	5.39

### Serological Assay and Data Analysis

The serological assay to detect specific antibody titers directed against the staphylococcal antigens was performed according to Meyer et al. (40). All antigens were presented on the surface of magnetic fluorophore-coded microspheres (MagPlex®/Luminex®, US) in a 79-plex assay. To achieve a high quality of data, seven serial dilutions spanning the range from 1:50 to 1:204,800 were analyzed for each serum. Human IgG and IgA specific detection antibodies labeled with R-Phycoerythrin were purchased from Jackson ImmunoResearch Europe Ltd (Ely, UK; order numbers 109-116-098 and 109-115-011). To retrieve a single characteristic value per serum/antigen from the multiple data points of the dilution series, the xMAPr analysis tool (40) was used for curve fitting and calculation of the response value. To avoid missing values in the data set, an imputation was performed to include cases where curve fitting failed based on a loess fit over all measurement of a single dilution. All statistical testings and data visualizations were performed using R [v4.0.2; (41)] together with Rstudio [v1.3.1056; (42)] and the tidyverse package [v1.3.0; (43)]. Data analysis was conducted separately for IgG and IgA, binary variables like *S. aureus* carriage or biological sex were addressed using the Wilcoxon signed rank test, reporting *p*-values after Benjamini-Hochberg correction (44) for multiple testing with a significance level of 0.05, whereas continuous and discrete variables like BMI or age were analyzed using spline interpolations. The spline regression analysis was performed using R (v4.0.1) in combination with tidyverse (v1.3.0) and limma (v3.44.3) packages. The phenotypes age, BMI or serum glucose were used as predictor variables for a natural cubic spline regression with four degrees of freedom, followed by a linear modeling and an empirical Bayes moderate *F*-test for each antigen, for the detection of general changes over the predictor variable for each antigen. The resulting *p*-values were adjusted using the Benjamini–Hochberg correction (44) and the significance level was set to 0.05.

## Results

Anti-staphylococcal antibody responses were detected in 996 individuals of the SHIP-TREND-0 cohort. The resulting data sets for IgG and IgA span a wide dynamic range due to the application of a dilution-based approach. The main findings are presented separately for IgG and IgA in the first and second section, with the combined findings following in the later sections.

### Analysis of Anti-staphylococcal IgG Titers

#### Overview on the Anti-*S. aureus* IgG Titers

**Figure 1:**
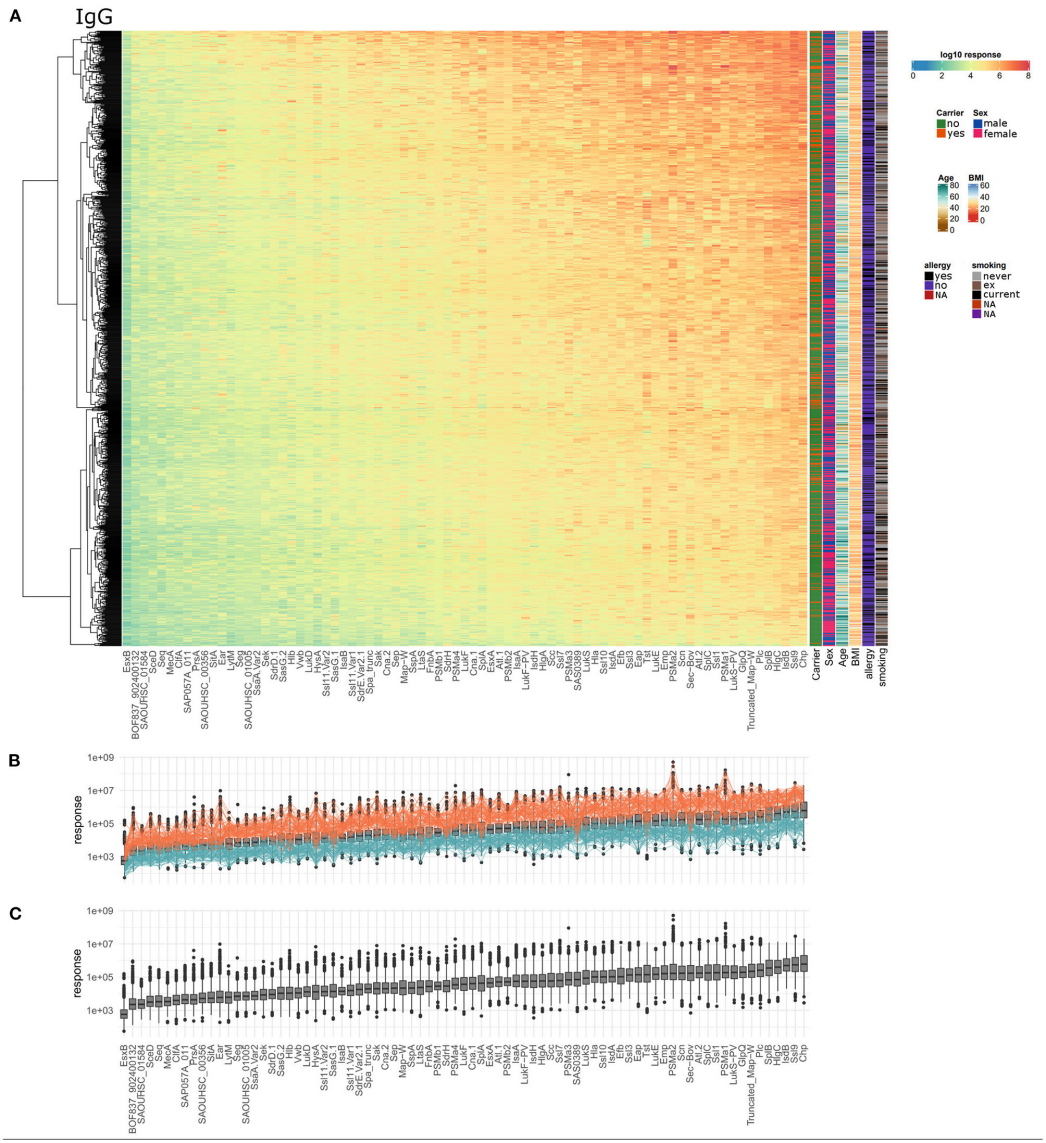
Serum IgG titers against *S. aureus* are highly variable with very large differences among the 996 tested individuals and also among the 79 included staphylococcal antigens. **(A)** The calculated IgG response values of all 996 individuals (y-axis) were plotted against the 79 antigens (x-axis) on a log10-transformed scale. Phenotypic information on the individuals is presented in additional columns on the right of the heatmap including carriage status, sex, age, BMI, allergy, and smoking. **(B)** Overview of overall high (orange) and overall low (petrol) responders, the calculated response is plotted as boxplot as in **(C)**. **(C)** Ranking of the antigens according to the median specific IgG levels.

Serum IgG titers against *S. aureus* antigens covered a very broad range with large differences between the 996 tested individuals as well as the 79 staphylococcal antigens. Every single individual harbored serum IgG antibodies against numerous *S. aureus* antigens. The wide dynamic range is depicted in Figure 1A as a heatmap, showing the calculated response values, which reflect antibody concentrations, for all antigens and all subjects. The dynamic range of the seven single dilutions included in the response calculation is shown in separate heatmaps in Supplementary Figures 1–7. The calculated antibody response values ranged from 5.5^*^10^1^ to 5.2^*^10^8^ for the 79 antigens spanning close to seven orders of magnitude. The average range of the response values per single antigen was 3.7 orders of magnitude. SAOUHSC_01584 showed the smallest span of about 2.5 orders of magnitude between minimal and maximal detected specific antibody response, while PSMα2 presented with the widest range of over 5 orders of magnitude (Figure 1C). Antibody responses against antigens did not cluster into distinguishably groups but are rather scattered over a large range of values. The antigen with the highest median antibody responses was CHIPS, followed by Ssl9, IsdB, HlgC, and SplB. The overall highest response value was acquired for PSMα2, one of the short peptide antigens of the group of phenol soluble modulins. SEQ, SceD, SAOUHSC_01584, BOF837_902400132, and EsxB make up the lower end in the ranking of all antigens according to the median antibody response.

A group of 60 individuals can be considered as “overall low responders” showing antibody response values in the lower third for more than 80% of the included antigens. On the other side, 66 individuals had antibody responses in the upper third of the range of each antigen for more than 80% of the antigens and can therefore be termed “overall high responders” (Figure 1B). Remarkably, the groups of high and low responders comprised <*7*% of the cohort each, reflecting the extraordinary diversity of the antibody binding patterns to the 79 staphylococcal antigens. Since the nasal *S. aureus* carriage status had been determined microbiologically during the medical examination of the probands, we know that 25.2% (251/996) of the individuals were colonized with *S. aureus* at the time of blood sampling, while 73.6% (733/996) were not carrying detectable amounts of *S. aureus* in the nose (39). For 12 individuals no carriage information was available. Non-carriers were strongly enriched among low responders (56 non-carriers, 3 carriers, 1 unknown) as compared to the high responders (42 non-carriers, 24 carriers) (Fisher's exact test resulting in *p* = 1.8^*^10^−5^).

#### Association of IgG Response Values With Specific Phenotypes

**Figure 2:**
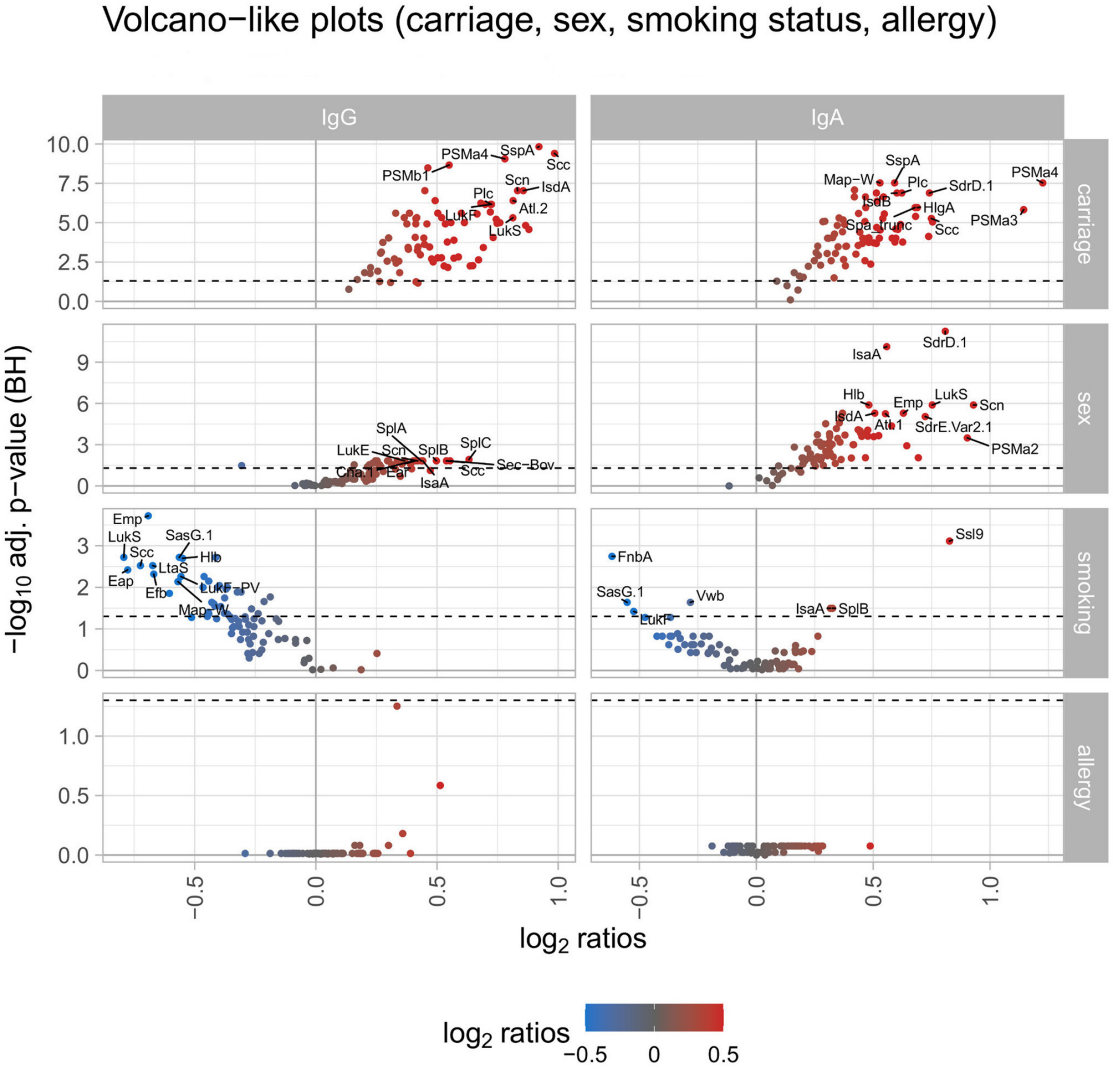
Volcano-like plots displaying the association of response values of antigens with carriage, sex, smoking, and allergy. For the categorical variables *S. aureus* carriage (carrier/non-carrier), sex (male/female), smoking (current smoker/non-smoker), and allergy (allergy/no allergy) the ratio was calculated between the means of each antigen and the *p*-value was derived from the Wilcoxon signed rank test. The Benjamini-Hochberg-adjusted *p*-values are depicted on the y-axis against the log2-ratios on the x-axis. The left column shows differences for IgG, the right column for IgA. The log2 ratio of 0 (no difference between the groups) is indicated as a solid vertical line. The significance threshold of 0.05 is indicated as a dotted line. The top 10 antigens showing the strongest associations in each comparison are labeled with the respective antigen's name. The color code displays associations from negative (blue) to positive (red).

Next, we correlated the IgG response values of each antigen with several phenotypes, i e., *S. aureus* carriage, sex, smoking status, allergies, age, BMI, and blood glucose levels to identify determinants for high antibody levels. Notably, *S. aureus* carriers (*n* = 251) presented a higher mean antibody response than non-carriers (*n* = 733) against each single *S. aureus* antigen (Figure 2, left panel showing a volcano plot of ratio and adjusted *p*-value; the full antigen-wise depiction of all ratios is shown in Supplementary Figure 8; details in Supplementary Table 2). The maximum ratio was detected for Scc, with a 1.98-fold higher mean antibody response in carriers. The median ratio of all 79 antigens was 1.40 between carriers and non-carriers. For 74 antigens this difference in mean antibody responses was significant (see detailed information on all addressed phenotypes in Supplementary Table 2).

A similar, though less pronounced effect was observed for males (*n* = 440) vs. females (*n* = 556). The mean antibody response for 68 antigens was higher in males than in females, for 26 antigens this difference was statistically significant. Of the 11 antigens with a higher mean IgG response in females, only one showed a significant result. To address the impact of smoking habits, the two extreme groups of current smokers (*n* = 219) vs. non-smokers (*n* = 419) were selected. The mean antibody response was higher in non-smokers for 75 antigens, a subgroup of 34 antigens reached statistical significance (Figure 2).

**Figure 3:**
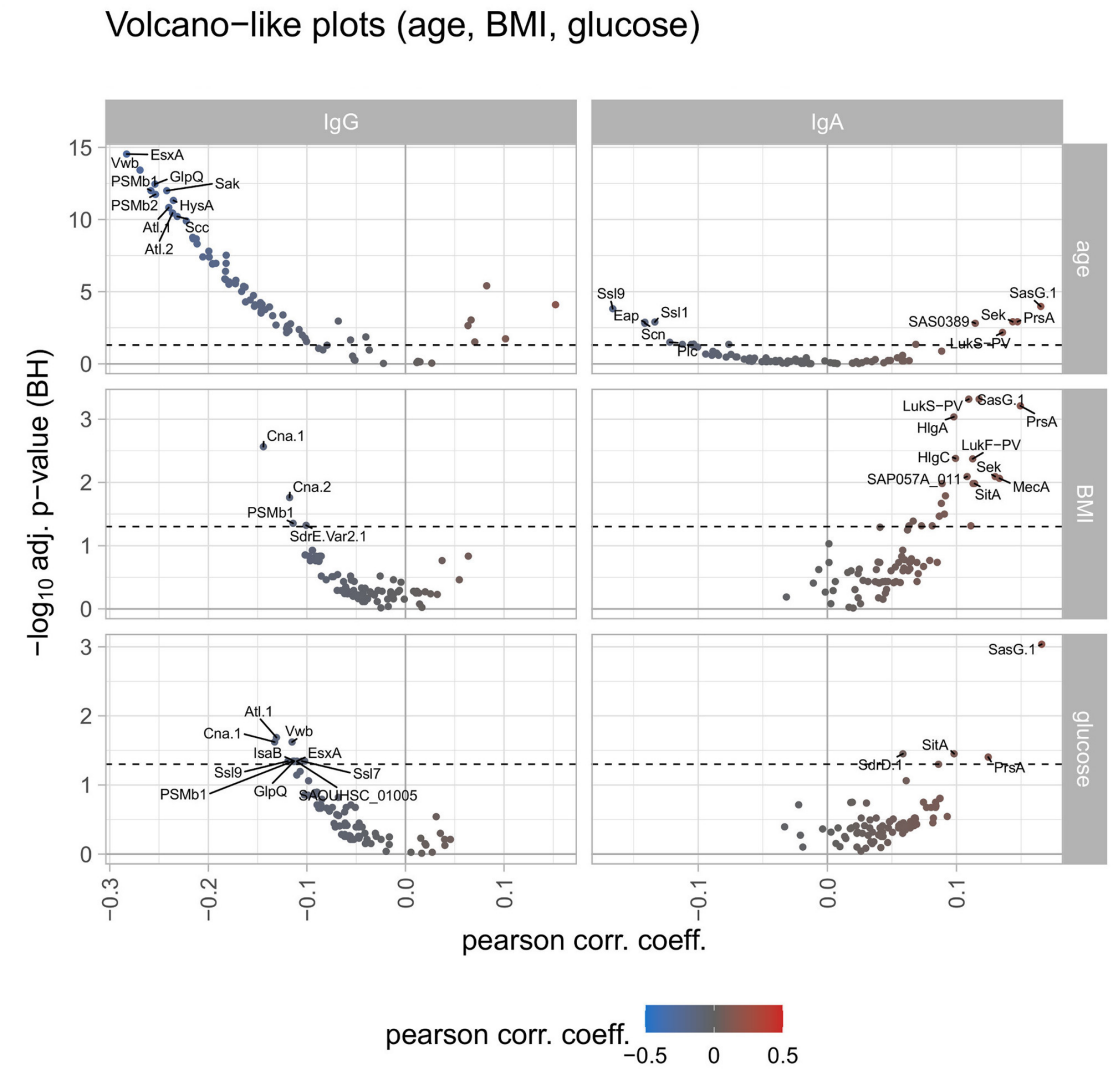
Volcano-like plots displaying the association of response values of antigens with age, BMI, and serum glucose. For the continuous variables age, BMI, and serum glucose levels a spline regression analysis was conducted followed by linear modeling to derive the Pearson correlation coefficient together with the *p*-value from the spline analysis. The Benjamini-Hochberg-adjusted *p*-values are depicted on the y-axis against the Pearson correlation coefficient on the x-axis. The left column shows differences for IgG, the right column for IgA. The log2 ratio of 0 (no difference between the groups) is indicated as a solid vertical line. The significance threshold of 0.05 is indicated as a dotted line. The top 10 antigens showing the strongest associations in each comparison are labeled with the respective antigen's name. The color code displays associations from negative (blue) to positive (red).

Correlating the antibody responses with the age of the study participants revealed declining anti-staphylococcal IgG values with increasing age for 69 antigens, with statistical significance for 61 antigens. The remaining 10 antigens showed an increase of antibody responses with increasing age with 6 of them reaching significance (namely SEQ, SEK, SasG.1, SAS0389, LukS-PV, LukF-PV). For BMI and serum glucose levels of the study participants the spline analysis resulted in similar findings: IgG binding to 66 antigens tended to decrease with increasing BMI as well as increasing serum glucose concentrations. Only two of those (namely Cna.1 and PSMb1) were significantly correlated within this group. For 10 antigens the antibody responses were increasing with the increase of BMI and serum glucose levels, the remaining 3 antigens showed trends in contrary directions for BMI and serum glucose levels (Figure 3).

Finally, we tested whether allergies influence the anti-*S. aureus* IgG response. Using information from the anamnestic interview of the SHIP-TREND-0 cohort, individuals were grouped according to the presence (*n* = 301) or absence (*n* = 694) of a medical diagnosis of allergy. While there were no significant differences between the two groups, 57 antigens trended to elicit higher and 22 antigens lower antibody responses in the allergic compared to the non-allergic individuals (Figure 2).

In summary, high anti-staphylococcal IgG responses were strongly associated with *S. aureus* carriage and male sex, and inversely correlated with age. Smoking, BMI and blood glucose levels moderately influenced the anti-staphylococcal serum IgG response, while allergies did not play a role.

### Analysis of Anti-staphylococcal IgA Responses

#### Overview on the Anti-*S. aureus* IgA Titers

**Figure 4:**
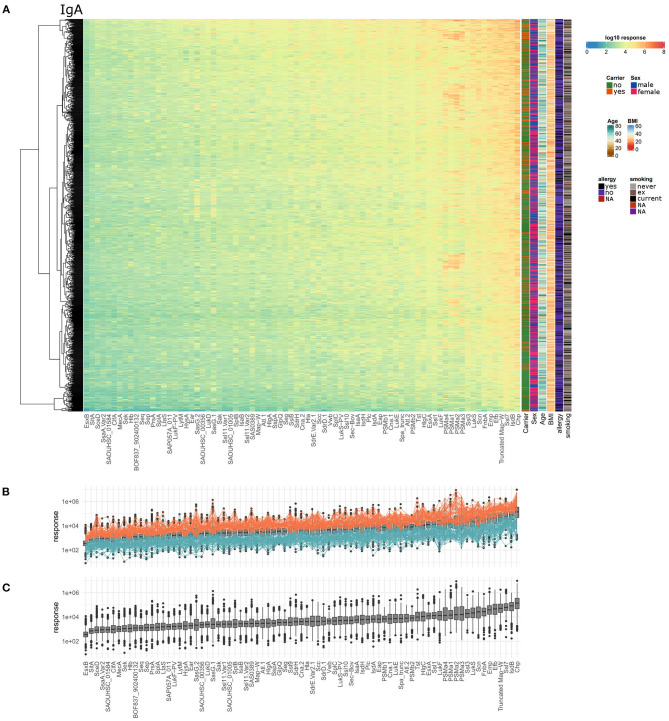
Serum IgA titers against *S. aureus* are highly variable with very large differences among the 996 tested individuals as well as the 79 included staphylococcal antigens. **(A)** The calculated IgA response values of all 996 individuals (y-axis) were plotted against the 79 antigens (x-axis) on a log10-transformed scale. Phenotypic information on the individuals was included in the additional columns on the right of the heatmap including carriage status, sex, age, BMI, allergy, and smoking. **(B)** Overview of overall high (orange) and overall low (petrol) responders, the calculated response is plotted as boxplot for all antigens and individuals, ordered with increasing median responses. **(C)** Ranking of the antigens according to IgA responses from all individuals.

Similar to the anti-staphylococcal IgG response, the IgA response was very heterogeneous with regard to the tested healthy individuals and staphylococcal antigens. Anti-staphylococcal serum IgA was detected in every single individual, but the spectrum of recognized antigens and the IgA response values differed strongly (Figure 4A). The IgA response values ranged from 8.0^*^10^0^ to 9.4^*^10^6^, covering about six orders of magnitude. The average range of antibody response values per antigen was 3.3 orders of magnitude, with GlpQ showing the smallest variability (2.4 orders of magnitude) and SasG.1 the highest (4.5 orders of magnitude). The top five antigens with the highest median IgA antibody response were CHIPS, IsdB, Ssl7, Truncated Map-W, and Efb. The antigens showing the lowest median IgA responses were SAOUHSC_01584, SsaA.Var2, SceD, SitA, and EsxB on the last position. The highest overall detected response value in the IgA data set was 9.4^*^10^6^ for CHIPS. The full data set is shown as a heatmap in Figure 4A, with the individual dilution plots included in Supplementary Figures 9–15.

Considering overall high and low responder groups for IgA, 59 individuals had response values in the upper third of the ranges for over 80% of the antigens (Figures 4B, 29 carriers, 29 non-carriers, 1 unknown). In one colonized individual, the antibody responses against all 79 antigens were within the upper third. At the lower end, 68 individuals had antibody response values in the lower third for more than 80% of the antigens (58 non-carriers, 9 carriers, 1 unknown). Thus, similar to the data obtained for IgG, *S. aureus* nasal carriers were overrepresented in the group of high responders (Fisher's exact test resulting in *p* = 0.0013).

#### Association of IgA Response Values With Specific Phenotypes

Since IgA responses are triggered under different conditions than IgG responses, we aimed to identify determinants for strong anti-staphylococcal serum IgA responses using the same phenotypes as in 3.1.2. Similar to the IgG response, the serum IgA response was higher in carriers than non-carriers for each tested antigen and reached statistical significance for 75 antigens (Figure 2, right panel; antigen-wise depiction of all ratios in Supplementary Figure 16; details in Supplementary Table 3). The ratio between carriers and non-carriers was the largest for two PSMs, namely PSMα4 and PSMα3, with a ratio of 2.33 and 2.21, respectively. The median ratio was 1.37 considering all 79 antigens. Again, in line with the IgG response, the mean IgA responses against 78 antigens were higher in males than females, 68 of those differences reached statistical significance.

In contrast to IgG, smoking habits were only weakly related to the anti-staphylococcal IgA response. Thirty-six antigens elicited higher IgA responses in current smokers than in non-smokers, whereas the opposite was true for 43 antigens. Three of the antibody responses with increased levels in smokers were tested significant, four of the antigens with higher responses in non-smokers showed significance as well (Figure 2).

Serum IgA antibody titers did not generally decline with advanced age, which was also different from IgG. For 52 antigens we detected decreasing mean antibody responses with increasing age, with 9 antigens reaching significance. The opposite trend was observed for the remaining 27 antigens, with 6 antigens showing a significant increase with age (namely LtaS, SAS0389, LukS-PV, SEK, PrsA, and SasG.1). Contrary to the IgG response, the IgA response values increased for most antigens with increasing BMI and serum glucose levels. A total of 71 antigens had higher antibody responses for IgA for both parameters, with 21 antigens reaching significance for BMI and 4 antigens for serum glucose levels (overlap: 3 antigens) (Figure 3). In terms of reported allergies, no statistically significant differences were observed for IgA antibody responses against the staphylococcal antigens included in this study (Figure 2).

### Integrated Analysis of IgG and IgA Data

#### Comparison of IgG vs. IgA High and Low Responders

Since IgG and IgA responses are triggered under different conditions (bacterial invasion vs. mucosal challenge), we hypothesized that the overlap between the groups of high IgG and IgA responders as well as that between low IgG and IgA responders should be small. Indeed, only 21 individuals belonged to the group of low responders for IgG (total: 60 individuals, see results 3.1.1) as well as for IgA (total: 68 individuals, see results 3.2.1). Even more striking, the overlap between the groups of high IgG and IgA responders comprised only 8 individuals (IgG high responders: 66 individuals; IgA high responders: 59 individuals). This suggests that IgG and IgA responses are indeed independent.

#### Comparison of IgG and IgA Response Values for the Different Staphylococcal Antigens

**Figure 5:**
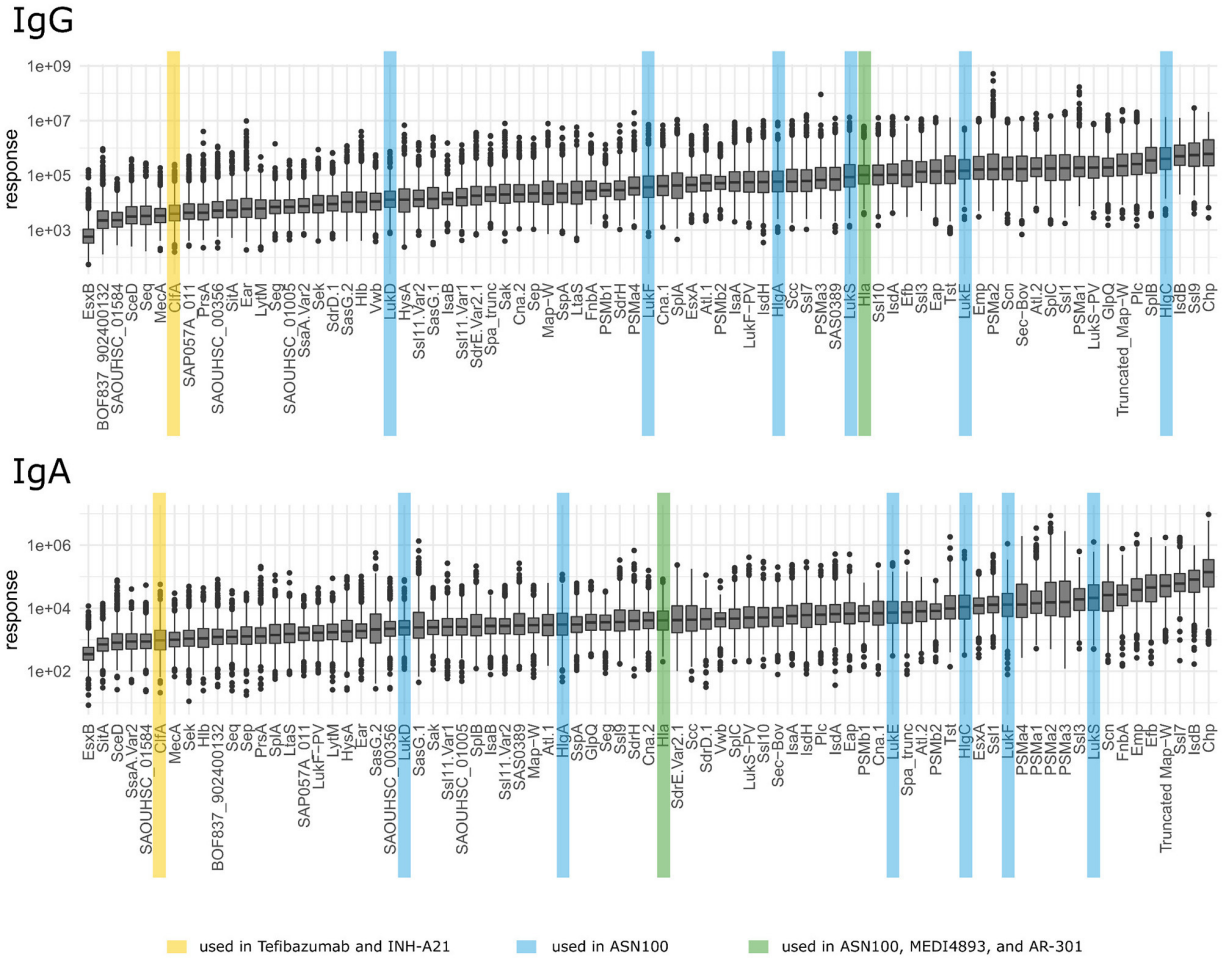
Ranking of antigens for IgG and IgA. Antigens used in vaccination studies before are highlighted by colors depending on the vaccine as explained in the bottom legend.

**Figure 6:**
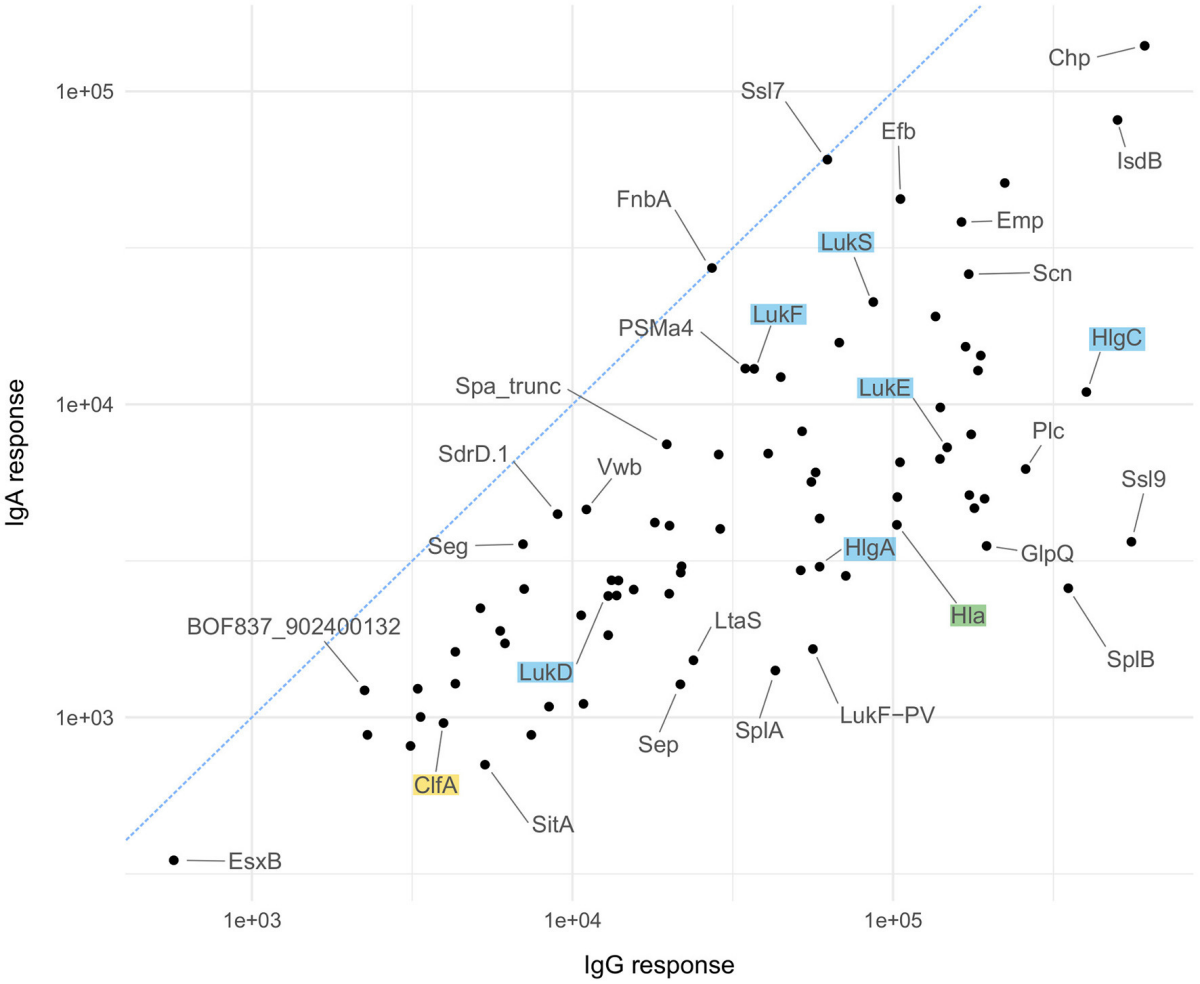
Antigen-wise comparison of IgG and IgA results. Comparison of median antibody responses between IgG (x-axis) and IgA (y-axis) for all 79 antigens. Candidates used in vaccination studies are highlighted in the same color scheme as explained for Figure 5.

As mentioned above, IgG and IgA responses reflect pathogen-host encounters in different micro-environmental niches where bacterial gene expression profiles and hence antigen release might differ strongly. Protein abundance, however, influences immunogenicity and antibody induction. Figure 5 depicts the ranking of the *S. aureus* antigens according to the median strength of antibody binding for IgG and IgA, those antigens already tested in vaccine trials are highlighted. In Figure 6 we compare the relative strength of antibody binding between IgG and IgA. CHIPS and EsxB make up the top and bottom end of the panel, with most antigens shifted toward higher median response values for IgG (below the blue dashed diagonal). SplB and Ssl9 elicited far more IgG than IgA, while in the case of FnbA and Ssl7, the IgA response was more prominent. A comparison of the ranking of all antigens can be found in Supplementary Table 4.

#### Comparison of Associations of IgG and IgA Response Values With Phenotypes

**Figure 7:**
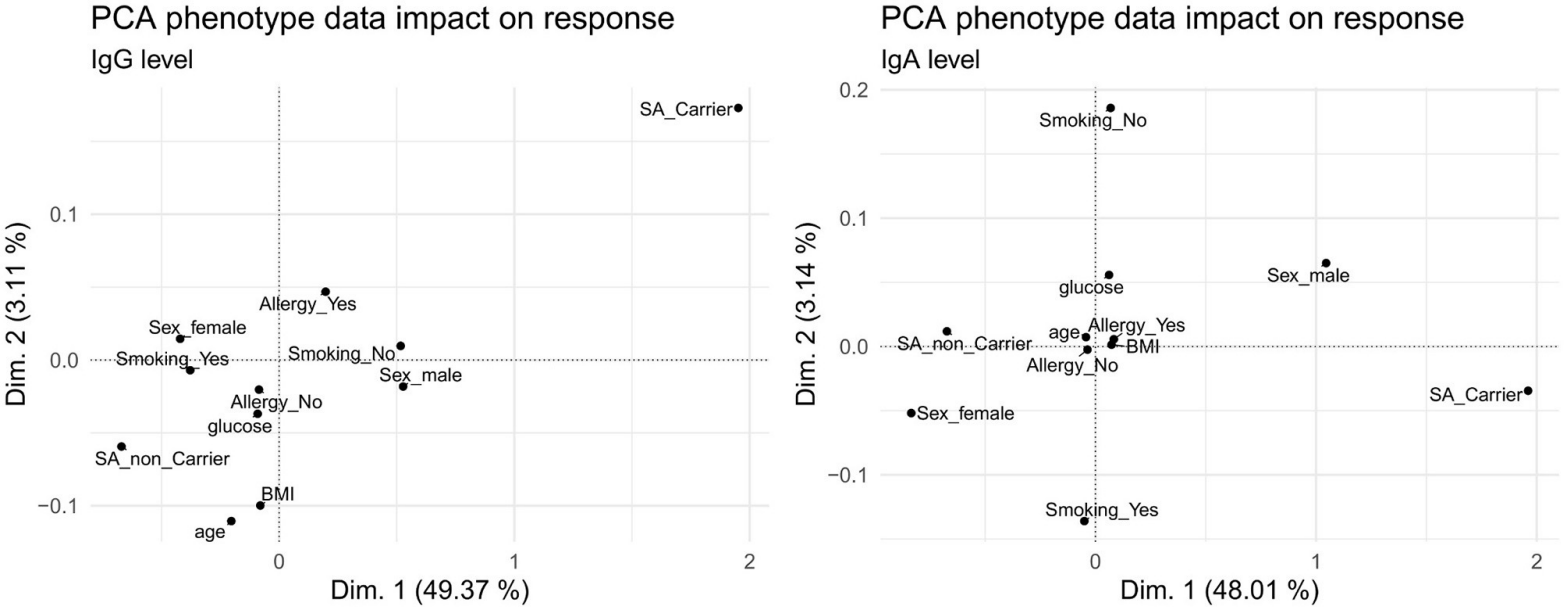
Principal component analysis of phenotype association with the overall IgG (left) and IgA (right) antibody response. In each panel, the first (x-axis) and second (y-axis) dimension are plotted against each other.

The IgG and IgA response values correlated with several phenotypic traits, with marked variations between the two antibody classes (Figures 2, 3). To visualize commonalities and differences more clearly, the association of phenotypes with the antibody levels over all antigens was summarized in a principal component analysis (PCA) shown in Figure 7 for the first and second dimension and Supplementary Figures 17, 18 until the fifth dimension. For both IgG and IgA, *S. aureus* carriage was identified as the main determinant, whereas sex showed a larger impact on IgA compared to IgG. Age had an influence on IgG, but not on IgA. Smoking, BMI, serum glucose, and allergy had only minor influence on both antibody classes. We further analyzed the impact of the associated phenotypes depending on the dimension of the PCA (results shown in Supplementary Figure 19), with carriage as main influencing factor in the first dimension for IgG and IgA, followed by age in the third dimension.

**Figure 8:**
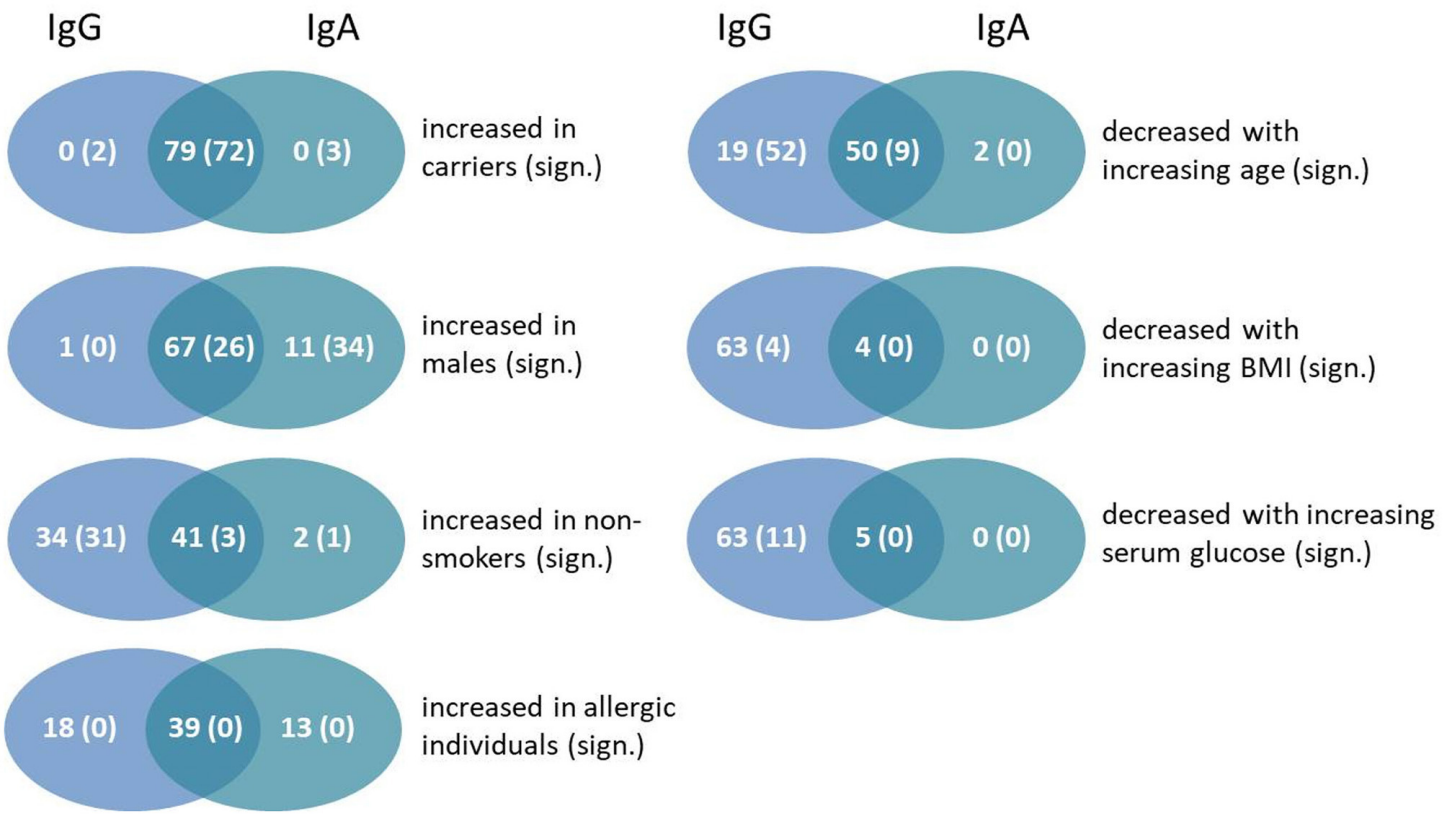
Venn diagrams showing the number and overlap of antigens showing an association of antibody responses values for IgG and IgA with particular phenotypes. The first number of each group gives the total amount of antigens with this characteristic, whereas the number in brackets includes only statistically significant numbers of antigens (Benjamini-Hochberg-adjusted *p*-value below 0.05).

Given the correlations of the IgG and IgA response with certain phenotypes (Figures 2, 3), we next asked whether these were due to the same antigens in both cases. The results are shown in the Venn diagrams in Figure 8. We found the strongest overlap when comparing carriers with non-carriers because antigen-specific IgG and IgA binding was always stronger in carriers. In 72 antigens the difference reached statistical significance for both antibody classes. For sex differences 67 antigens showed increased responses in males for IgG and IgA, but with more significant results on the side of IgA. The opposite direction was found for increased responses in non-smokers with far more antigens showing statistically significant results for IgG compared to IgA. As allergy did not correlate with antibody binding to the bacterial antigens, there were no significant differences at all. Age, the BMI as well as serum glucose seemed to influence IgG more strongly than IgA; in all cases there was an inverse correlation between the phenotype parameters and the strength of antibody binding.

### Influence of Strain-Specific Genomic Features on the Antibody Response of Carriers

We previously reported that *S. aureus* carriers mount a strain-specific antibody response against the superantigens produced by their colonizing strain with limited cross-reactivity to other superantigens (22). To re-evaluate this observation in our cohort, we took advantage of the genetic information that was available for a subset of 240 colonizing *S. aureus* isolates from this study cohort, which comprised the *spa* type, clonal complex, and presence of genes coding for the superantigens TSST-1 and the staphylococcal enterotoxins (39). To test for strain-specific antibody responses we compared anti-superantigen IgG and IgA profiles in individuals colonized with strains harboring or lacking the corresponding superantigen genes.

Carriers of *tst*-encoding *S. aureus* isolates (*n* = 48) showed a significantly higher IgG and IgA response against TSST-1 and also SEP than carriers of *tst*-negative isolates (*n* = 192) (Supplementary Figure 20). Similarly, individuals colonized with *S. aureus* strains harboring the pathogenicity island (SaPI1)-encoded superantigen genes *sek* and/or *seq* (*n* = 7 for *sek*/*seq* and *n* = 2 for *seq* alone) showed higher antibody responses for SEK, SEQ, and Ear, with Ssl1 additionally for individuals with *seq*-positive strains.

Surprisingly, there was no correlation between the occurrence of the *sep* gene and the SEP-specific antibody titers. This might be explained by a discrepancy between the *sep* allele amplified with our PCR and that encoding the recombinant SEP protein used for the measurement of antibody binding. The primer sequence for the genetic analysis was derived from strain *S. aureus* N315 (45), while the *sep* sequence of strain T0131 was used for recombinant protein expression. The two SEP-variants have only 77% sequence identity. Comparing the antibody titers of carriers colonized with strains equipped with *sep* to those without the gene the former showed significantly increased levels of antibodies against Ssl6 (SAS0389) and BOF837_902400132 for IgG and IgA, and additionally SplA, SplB, and SplC for IgA only.

## Discussion

### Characteristics of This Study

This study provides a comprehensive overview on the *S. aureus*-specific antibody repertoire in a large population-based study, SHIP-TREND-0. Using a dilution series approach rather than single-point measurements allowed us to precisely quantify anti-staphylococcal antibodies in both low and high titer sera and to detect subtle differences [for details on the used method see (40)]. We mainly focused on extracellular antigens of *S. aureus* including both secreted and surface-associated proteins, since these are accessible to the humoral immune system when facing live bacterial cells and hence induce a strong antibody response (25). Antigens were derived from *S. aureus* NCTC8325 only, but there are numerous allelic variants in the species *S. aureus*. Studies of superantigens illustrate that the antibody response to *S. aureus* virulence factors has a strain-specific component (22, 46). However, superantigens are an extreme example of antigenic variability in *S. aureus* that exceeds allelic variation. Most antigens are more conserved than superantigens, and allelic variation usually affects only a small portion of an antigens. Thus, conserved antigens and epitopes likely dominate the (polyclonal) antibody response to *S. aureus* and therefore, the analysis of antigens from a selected, well-established strain, while not perfect, provides a reliable estimate of the immunodominance of *S. aureus* antigens.

Using available meta data we could pinpoint multiple factors correlating with the strength of the anti-staphylococcal antibody response in naturally exposed humans. Comparing our results to earlier studies, we corroborate the large inter-individual differences in the anti-staphylococcal antibody profiles that cannot be tracked down to single factors (36). Nevertheless, we report several factors such as smoking habits that have not been directly linked to reduced antibody levels until now, or BMI and serum glucose showing only small but distinct differences. Combining our antibody data with the genetic background information on the colonizing *S. aureus* strains we could show the impact of single staphylococcal antigens on the antibody response in individuals whose immune system is confronted with these proteins on a regular basis.

### Heterogeneity of Antibody Titers and Their Potential Clinical Impact

Both serum IgG and IgA titers against *S. aureus* were highly heterogeneous. Anti-staphylococcal IgG and IgA were detected in every single individual, albeit in highly variable quantity and composition. In this respect, our data confirm and extend previous studies (e g., 36) because we analyzed a large set of antigens (*n* = 79) and many individuals (*n* = 996). The highly individual antibody repertoire likely reflects the history of encounters with *S. aureus*. Indeed, we observed that carriers mount a specific response against the superantigens of their colonizing strains (namely TSST-1, SEK, and SEQ), which is in line with our previous observations (22). However, genetic variation between strains can make it hard to track down such correlations, as shown for SEP.

Using our highly sensitive multiplex approach, we were able to detect antibodies against all tested antigens. This emphasizes the ubiquitous nature of *S. aureus*. Likely, every individual encounters numerous *S. aureus* strains with very diverse antigen repertoires throughout their lifetime. This infers that even persistent *S. aureus* carriers are exposed to different *S. aureus* isolates, either by consecutive colonization with different isolates over the years, by co-colonization with multiple strains, or by infection with exogenous *S. aureus* strains (17, 46). In addition, antibody binding could reflect cross-reactivity to closely related antigens such as superantigens (47) and pore-forming toxins.

Even though anti-*S. aureus* antibodies obviously do not eliminate *S. aureus* from the nasal mucosa and do not reliably protect from infection, there is growing evidence that a strong immune memory, as reflected by high antibody titers, protects from severe *S. aureus* disease (23–25). Our data clearly show that around 6% of the tested individuals were low responders with antibody response values in the lower third of the binding range for more than 80% of the antigens. Based on our current knowledge, these individuals would be prone to a severe disease course in case of *S. aureus* infection. Tailored preventive or therapeutic measures, e g., decolonization, intravenous immunoglobulins (IVIG), and monoclonal antibodies could improve the outcome of such vulnerable patients in the future (48, 49).

The protective potential of anti-staphylococcal antibodies has been most convincingly shown for the superantigen-driven toxic shock syndrome (TSS). Anti-TSST-1 antibodies are highly prevalent in the Caucasian population (about 90%). Only people without detectable anti-TSST-1 antibodies are at risk of TSS, and symptoms can be treated by IVIG containing neutralizing antibodies (50, 51). Using the multiplex approach with seven serial dilutions of serum samples, we detected antibodies against TSST-1 in all samples (response values spanning from 7.5^*^10^2^ to 1.3^*^10^7^), pointing toward a higher assay sensitivity as compared to the ELISA used in earlier studies (51). This suggests that very low anti-TSST-1 antibody responses might not confer protection.

The quality of the antibody response to *S. aureus* depends on the nature of the encounter with the bacteria. Antibody responses in the mucosa are governed by IgA, with local mucosal titers being reflected in the systemic availability of specific monomeric IgA (28, 36), while invasive infections are controlled by IgG. This dichotomy is also reflected by our results. The ranking of antigens based on the median antibody response was similar for only 28 antigens, suggesting that different gene expression profiles are executed during mucosal encounters vs. systemic infection. Similarly, others also observed limited overlap in the anti-staphylococcal IgG and IgA response in healthy carriers and non-carriers (30, 36). CHIPS ranked first for both IgG and IgA as previously reported (36), implying a high immunogenicity of this immune evasion factor. Moreover, the overlap between high IgG vs. IgA responders was limited, suggesting that these immune response modes are indeed independent.

In this study, we used serum IgA as a surrogate marker for mucosal IgA production, because nasal secretions were not available. This approach is supported by growing evidence for a clonal relationship of serum IgA and mucosal IgA responses. For example, proteomic analyses of a celiac disease cohort identical antigen-binding regions of gluten-specific serum IgA and mucosal IgA (secreted by gut-derived plasma blasts) (28). This suggests that serum and mucosal IgA originate from the same B cell clones but are produced by individual plasmablasts in different locations. Indeed, several studies demonstrate that B cells activated in the gut immune system give rise to plasma cells that reside in the lamina propria and produce dimeric IgA for transport across the epithelium as well as an equivalent population of plasma cells that migrate to the bone marrow and secrete monomeric IgA into the circulation (28, 52–54). Hence, serum monomeric IgA can serve as a proxy for the hardly accessible secretory dimeric IgA of mucosae (28). In line with this, it has already been shown by Verkaik et al. (36) that for staphylococcal antigens serum antibody titers of IgG and IgA can be correlated to those in the nasal secretion.

### Colonization With *S. aureus* Strongly Impacts on the Anti-staphylococcal Antibody Response

Carrying *S. aureus* as part of a healthy human microbiome has long been suspected to increase the amount of anti-staphylococcal antibodies due to repeated minor invasive episodes caused by the colonizing strain (36). With our data we support this notion. All antigen-specific antibody responses were stronger in carriers than in non-carriers, in most cases significantly. With ratios of 1.10–1.98 between the mean responses of the carrier group vs. the non-carrier group the direction of the trend is very clear. However, the effect size of carriage was minute compared to the variability of the individual antibody response to each antigen.

### Sex and Age Are Correlated With the Anti-staphylococcal Antibody Response

Men had on average higher anti-*S. aureus* antibody levels than women, both IgG and IgA. This is unusual because generally women produce higher antibody levels than men. Klein and Flanagan reviewed multiple features of immunity in humans influenced by biological sex (55). Combining the results of many studies, they concluded that regardless of age, females tend to show stronger antibody responses than males, with higher basal levels of immunoglobulin and increased B cell numbers (55). The more pronounced antibody response to *S. aureus* antigens in men could be explained by their more extensive exposure to the bacteria. Using culture-based detection methods, men tended to have higher colonization rates than females (12). DNA sequencing, however, revealed overall higher colonization rates (53%) that were equal for men and women (13). This discrepancy can be explained by a 10–100-fold higher absolute abundance of *S. aureus* in men and its influence on culture outcomes. This higher bacterial load is probably the main reason for the consistently higher anti-staphylococcal antibody levels in men. In our study cohort we reported a culture-based carriage rate of 27.0 vs. 24.3% for males and females, respectively, but according to Liu et al. (13) we might have failed to detect carriers with low bacterial loads, especially among female study participants (39).

Only the IgG responses consistently decreased with age. Regarding IgA, there was no clear trend; antibody binding to two thirds of the antigens decreased with age, the remainder showed the opposite trend. The decline in specific IgG titers could reflect a gradual decline of the adaptive immune functions with age, called immunosenescence. It diminishes the host's ability to clear pathogens and also to develop long-term memory to vaccination. This results in a steady increase of the incidence of infections with age in the elderly. In the humoral immune system alterations in the B cell repertoire and subcompartment distribution, as well as defects in B lymphopoiesis, cell development and homeostasis are observed. These lead to reduced pathogen-specific antibodies in the elderly [reviewed in (56, 57)]. Since age-dependent analyses of the B cell response and antibody repertoires against *S. aureus* have not been performed yet, it remains to be shown which features of immune aging are responsible for the decline in *S. aureus*-specific antibody levels. *S. aureus* colonization rates also decrease with increasing age in humans, which is counterintuitive considering the decreasing antibody levels. A noteworthy exception from this general trend are two matching candidate antigens namely LukF-PV and LukS-PV where antibody titres increased with age. The genes for the pore-forming toxin Luk-PV are very rare in colonization strains in our cohort; they are carried by only 0.1% of the nasal isolates. Probably age groups face different strains because of their different lifestyle; e g., contacts at work or in health care facilities. Long-term observational studies correlating the changes in the antibody repertoire with challenges by various strains with distinct sets of mobile genetic elements are required to assess the impact of such factors.

### Influence of Further Factors on the Anti-staphylococcal Antibody Response

Besides sex and age, we included additional lifestyle factors in our study, revealing even more pieces of influence on the anti-staphylococcal antibody repertoire in the puzzle of individual variability. Obesity, chronic diseases of the skin (e g., atopic eczema), but also smoking habits have been shown to alter colonization rates (7, 11, 14). With our data we could show that BMI, elevated serum glucose, and regular smoking impact on the antibody levels against staphylococcal antigens, while medically diagnosed allergies did not play a role.

Elevated BMI and serum glucose levels were inversely correlated with antibody responses against most of the included antigens. This may be due to the impaired function of the adaptive immunity in obese people already described in the context of vaccination against hepatitis and influenza [reviewed in (58)]. We found the opposite trend in our IgA measurements: antibodies to most antigens increased with increasing BMI or serum glucose. This falls in line with the observation of a higher *S. aureus* colonization rate in obese individuals and patients with diabetes than in healthy individuals (59). The higher challenge to the immune system of the nasopharyngeal mucosa could trigger more IgA secretion.

Smoking has been described to alter the nasal microbiome and decrease the *S. aureus* colonization rate in humans (14). Interestingly smoking was associated with lower specific IgG levels, while for IgA no clear trend could be observed. Higher anti-Ssl9 and lower anti-FnbA levels in current smokers were sticking out. Obviously, there must be other factors besides colonization that influence the systemic (IgG) and local (IgA) immune response to *S. aureus*.

### Lessions Learned for Vaccine Development

Our analyses have implications for vaccine development against *S. aureus*. The selection of vaccine targets needs to consider their relevance in different staphylococcal diseases, prevalence in clinical isolates but also the pre-existing immunity in the population and the quality of the immune response that is generated during infection. Judging by the broad range of the median antibody response values observed in this healthy cohort that is spanning several orders of magnitude, selecting vaccine targets with a low pre-existing response may improve vaccine efficacy. Notably, past and current vaccine candidates strongly differ in their natural immunogenicity, with rather low antibody levels against ClfA (passive vaccine Tefibazumab) (60), and intermediate to high antibody levels against pore-forming toxins (Hla, HlgBC, LukED, LukSF, LukS-PV) (34, 61). However, the huge interindividual variation in antibody titers that cover several orders of magnitude suggests that a considerable proportion of individuals lacks protective antibody levels even against the highly immunogenic bacterial proteins. These would benefit from vaccination. Besides active vaccination, passive vaccination approaches might help to overcome this problem in the future: monoclonal antibody cocktails could be individualized by factoring in the virulence factor repertoire of the invasive strain as well as the antibody profile of the patient.

## Data Availability Statement

The raw data supporting the conclusions of this article will be made available by the authors, without undue reservation.

## Ethics Statement

The studies involving human participants were reviewed and approved by The Clinical Ethics Committee University Medicine Greifswald, Greifswald, Germany. The patients/participants provided their written informed consent to participate in this study.

## Author Contributions

TM, SH, FS, HV, BB, and UV: conceptualization. TM, SM, SH, NF, TK, and CK: data generation. TM, SM, SH, SW, NF, BB, and UV: data analysis. TM, SM, SH, BB, and UV: writing-original draft. TM, SM, SH, SW, NF, HV, TK, CK, FS, BB, and UV: writing-review and editing. BB and UV: project administration and funding. All authors: have read and agreed to the published version of the manuscript.

## Conflict of Interest

The authors declare that the research was conducted in the absence of any commercial or financial relationships that could be construed as a potential conflict of interest.
