# Supplementary material for: A Comprehensive View on the Human Antibody Repertoire Against Staphylococcus aureus Antigens in the General Population
Source: Front Immunol. 2021 Mar 10;12:651619. doi: 10.3389/fimmu.2021.651619 (PMC7987813; doi:10.3389/fimmu.2021.651619)
Supplement: Supplementary file 5 [file Data_Sheet_1.docx]

Supplementary Material

# Supplementary Figures
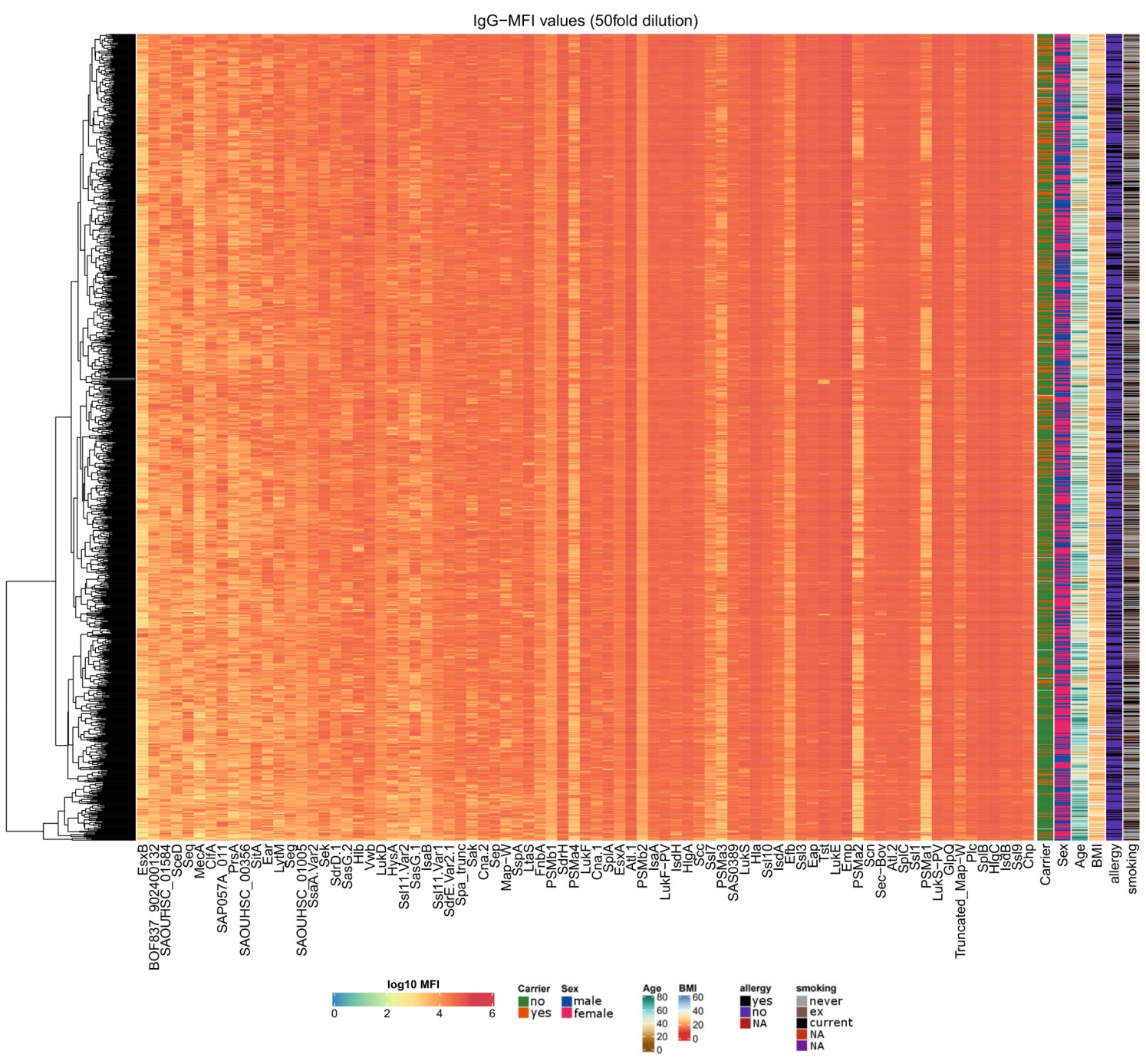


**Supplementary Figure 1.** The median fluorescense intensities in the IgG measurement of the 50-fold dilution of all 996 individuals (y-axis) were plotted against the 79 antigens (x-axis) on a log10-transformed scale. Phenotypic information on the individuals was included in the additional columns on the right of the heatmap including carriage status, sex, age, BMI, allergy, and smoking.


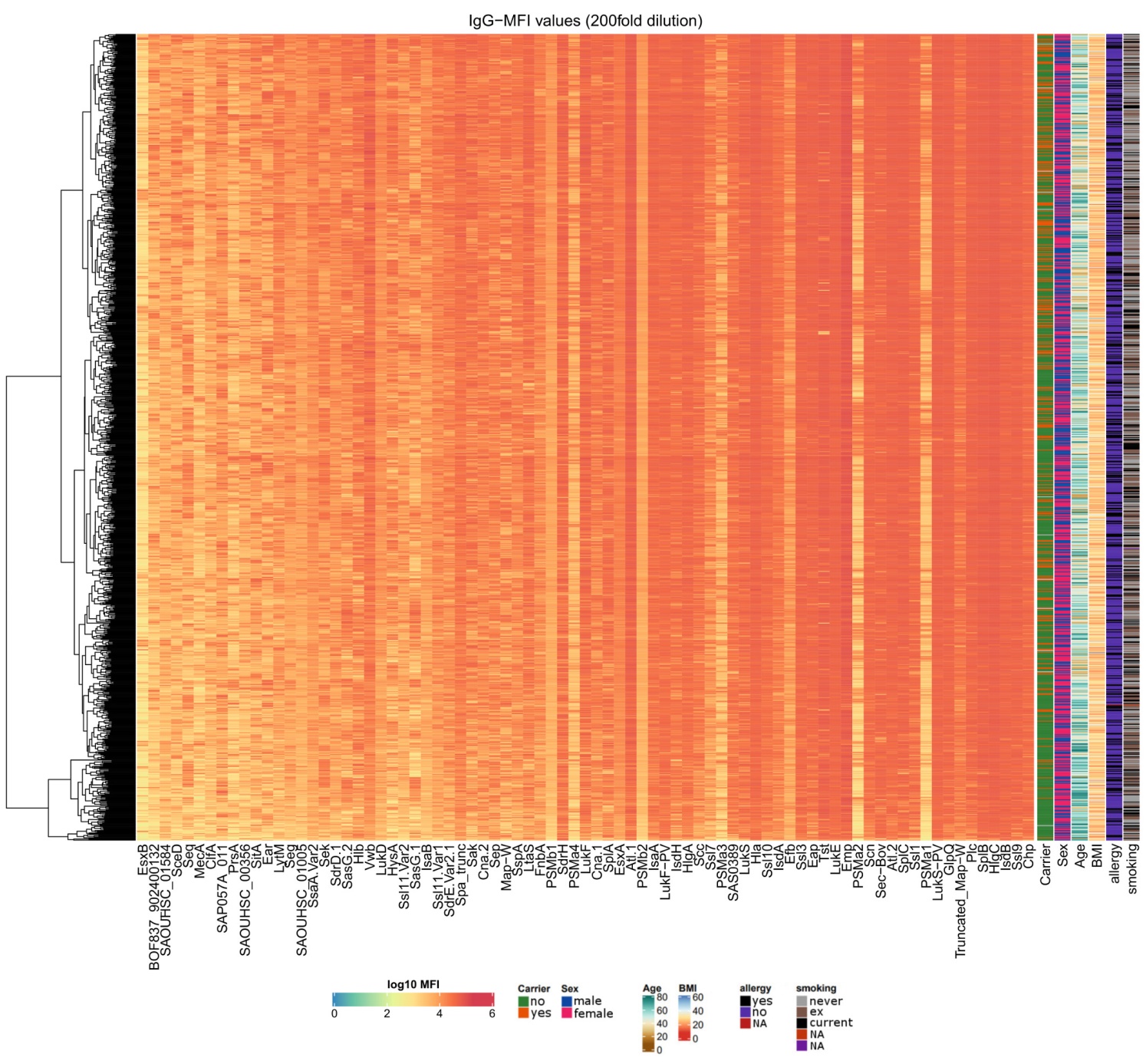


**Supplementary Figure 2.** The median fluorescense intensities in the IgG measurement of the 200-fold dilution of all 996 individuals (y-axis) were plotted against the 79 antigens (x-axis) on a log10-transformed scale. Phenotypic information on the individuals was included in the additional columns on the right of the heatmap including carriage status, sex, age, BMI, allergy, and smoking.


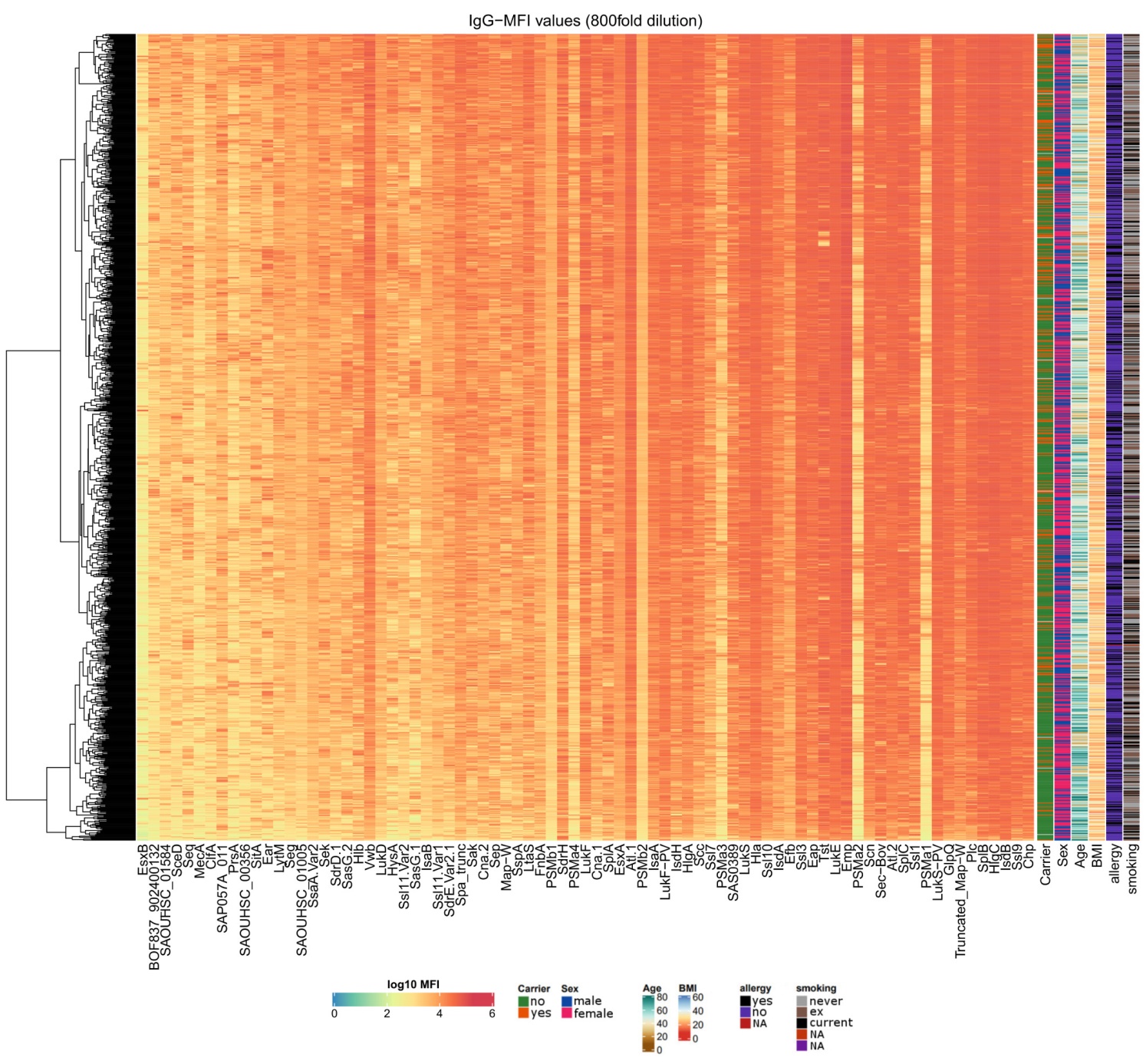


**Supplementary Figure 3.** The median fluorescense intensities in the IgG measurement of the 800-fold dilution of all 996 individuals (y-axis) were plotted against the 79 antigens (x-axis) on a log10-transformed scale. Phenotypic information on the individuals was included in the additional columns on the right of the heatmap including carriage status, sex, age, BMI, allergy, and smoking.


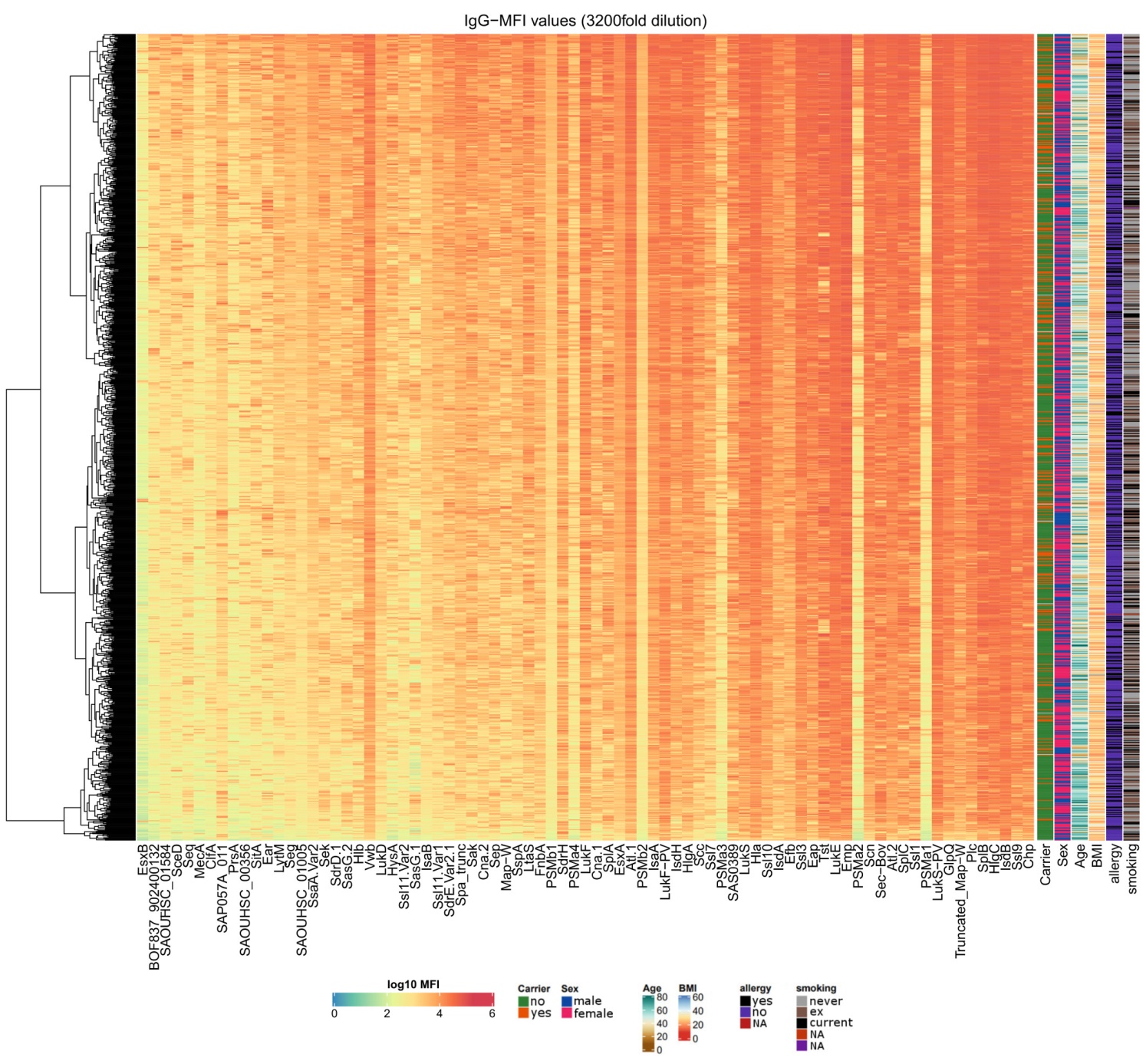


**Supplementary Figure 4.** The median fluorescense intensities in the IgG measurement of the 3,200-fold dilution of all 996 individuals (y-axis) were plotted against the 79 antigens (x-axis) on a log10-transformed scale. Phenotypic information on the individuals was included in the additional columns on the right of the heatmap including carriage status, sex, age, BMI, allergy, and smoking.


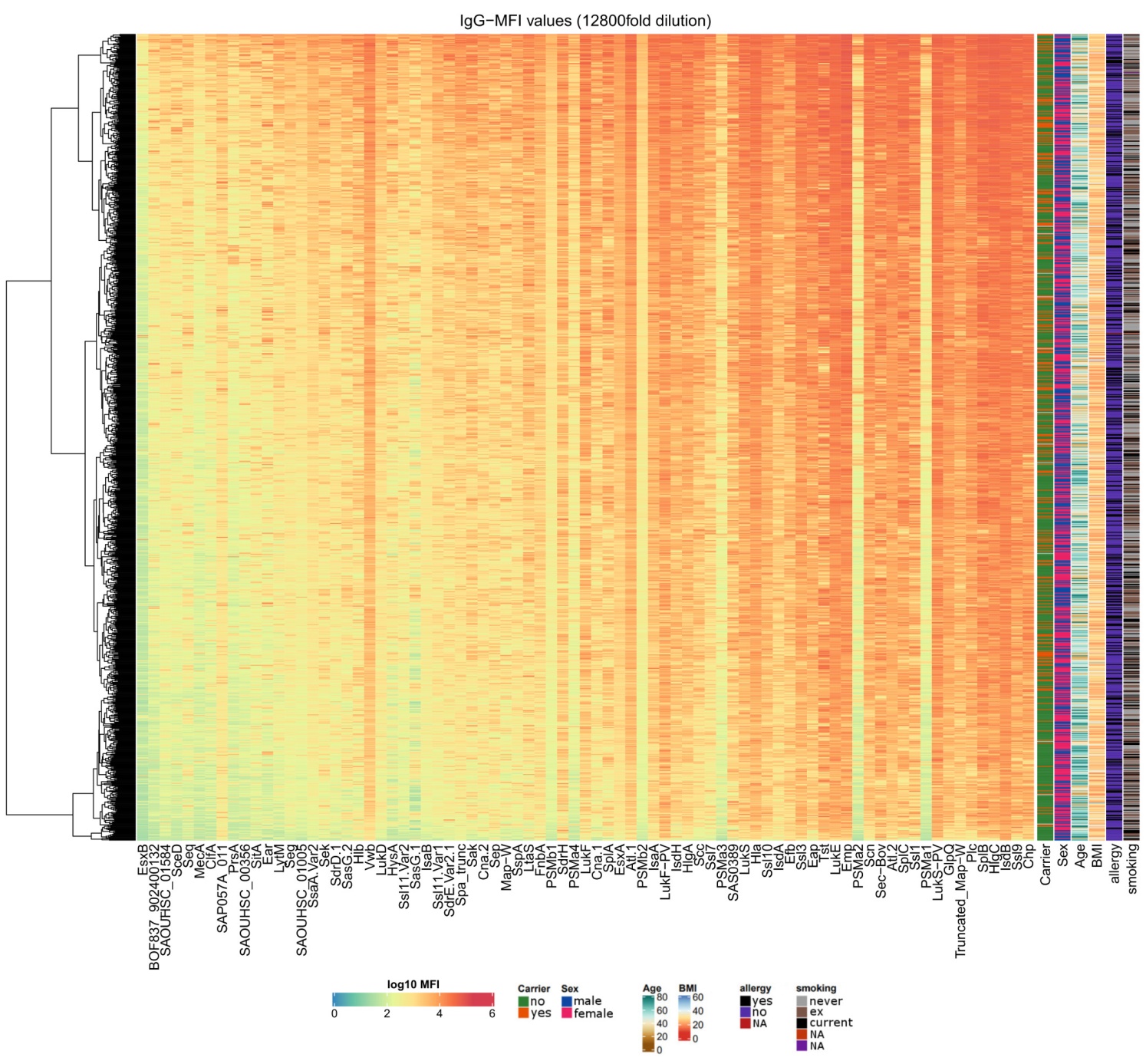


**Supplementary Figure 5.** The median fluorescense intensities in the IgG measurement of the 12,800-fold dilution of all 996 individuals (y-axis) were plotted against the 79 antigens (x-axis) on a log10-transformed scale. Phenotypic information on the individuals was included in the additional columns on the right of the heatmap including carriage status, sex, age, BMI, allergy, and smoking.


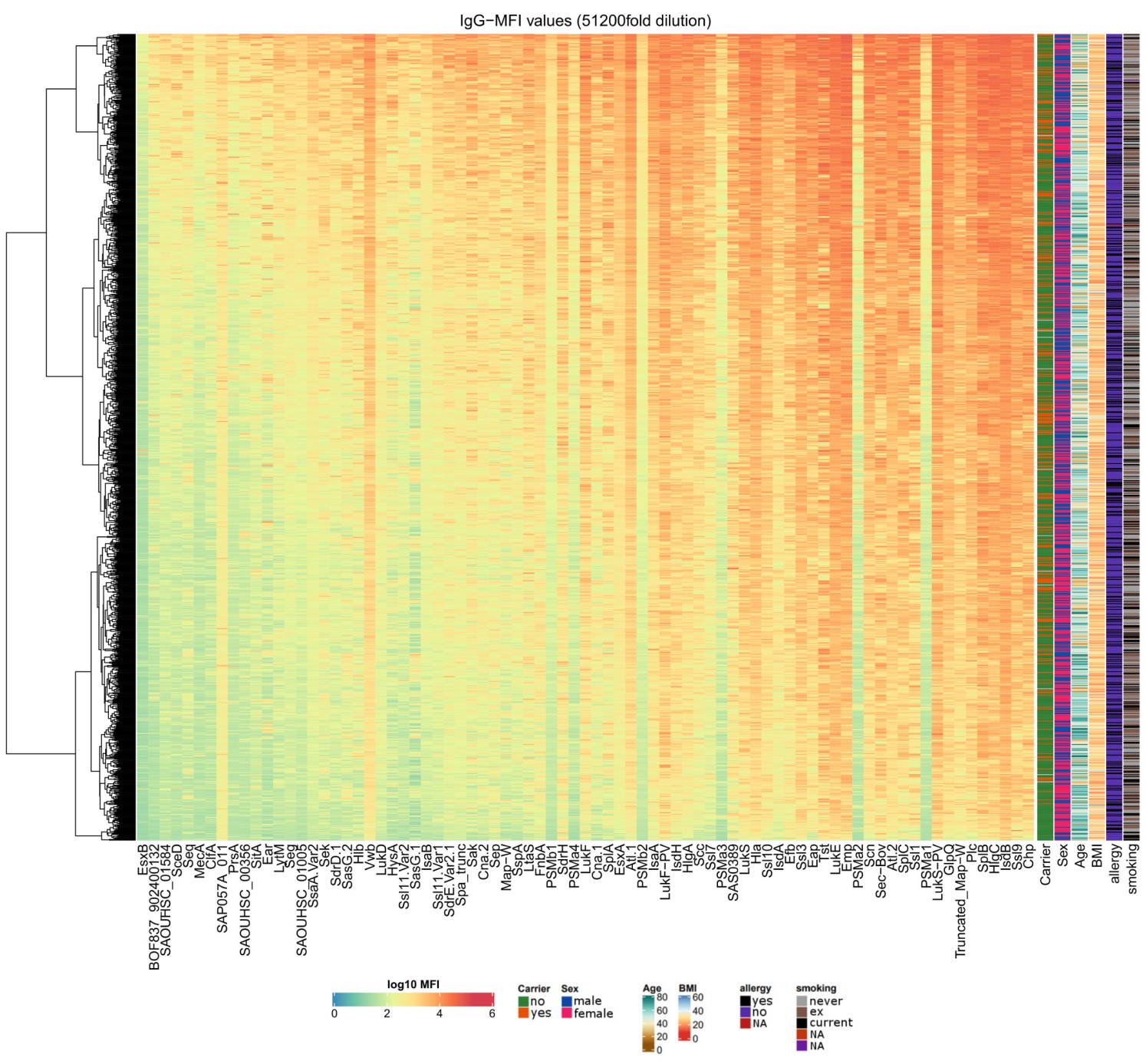


**Supplementary Figure 6.** The median fluorescense intensities in the IgG measurement of the 51,200-fold dilution of all 996 individuals (y-axis) were plotted against the 79 antigens (x-axis) on a log10-transformed scale. Phenotypic information on the individuals was included in the additional columns on the right of the heatmap including carriage status, sex, age, BMI, allergy, and smoking.


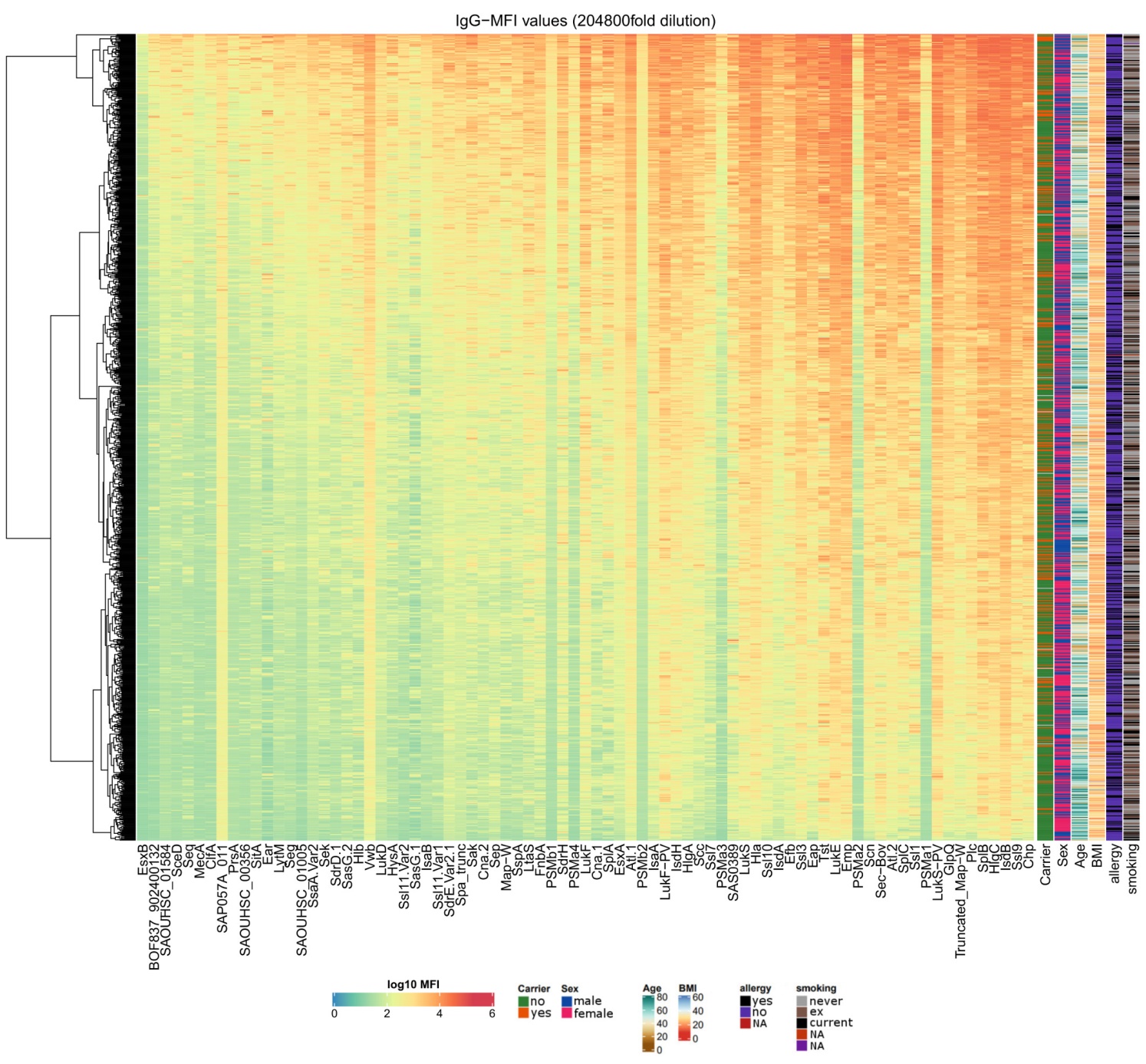


**Supplementary Figure 7.** The median fluorescense intensities in the IgG measurement of the 204,800-fold dilution of all 996 individuals (y-axis) were plotted against the 79 antigens (x-axis) on a log10-transformed scale. Phenotypic information on the individuals was included in the additional columns on the right of the heatmap including carriage status, sex, age, BMI, allergy, and smoking.


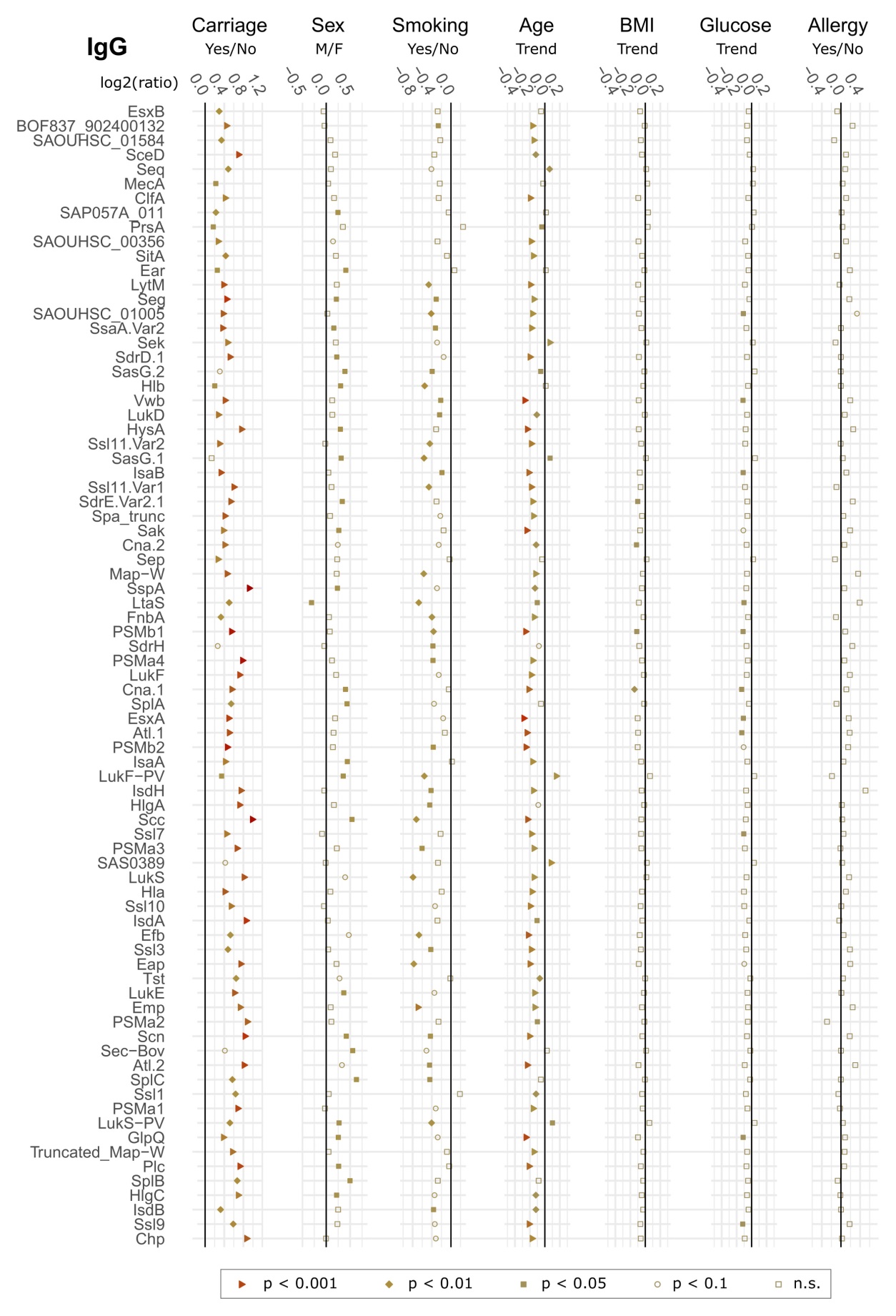


**Supplementary Figure 8.** Antigen-wise depiction of IgG derived ratios from group comparisons. The log2 ratio is shown on the x-axis in each panel for all 79 antigens on the y-axis. The significance value of each comparison is encoded in color and shape as shown in the bottom legend.


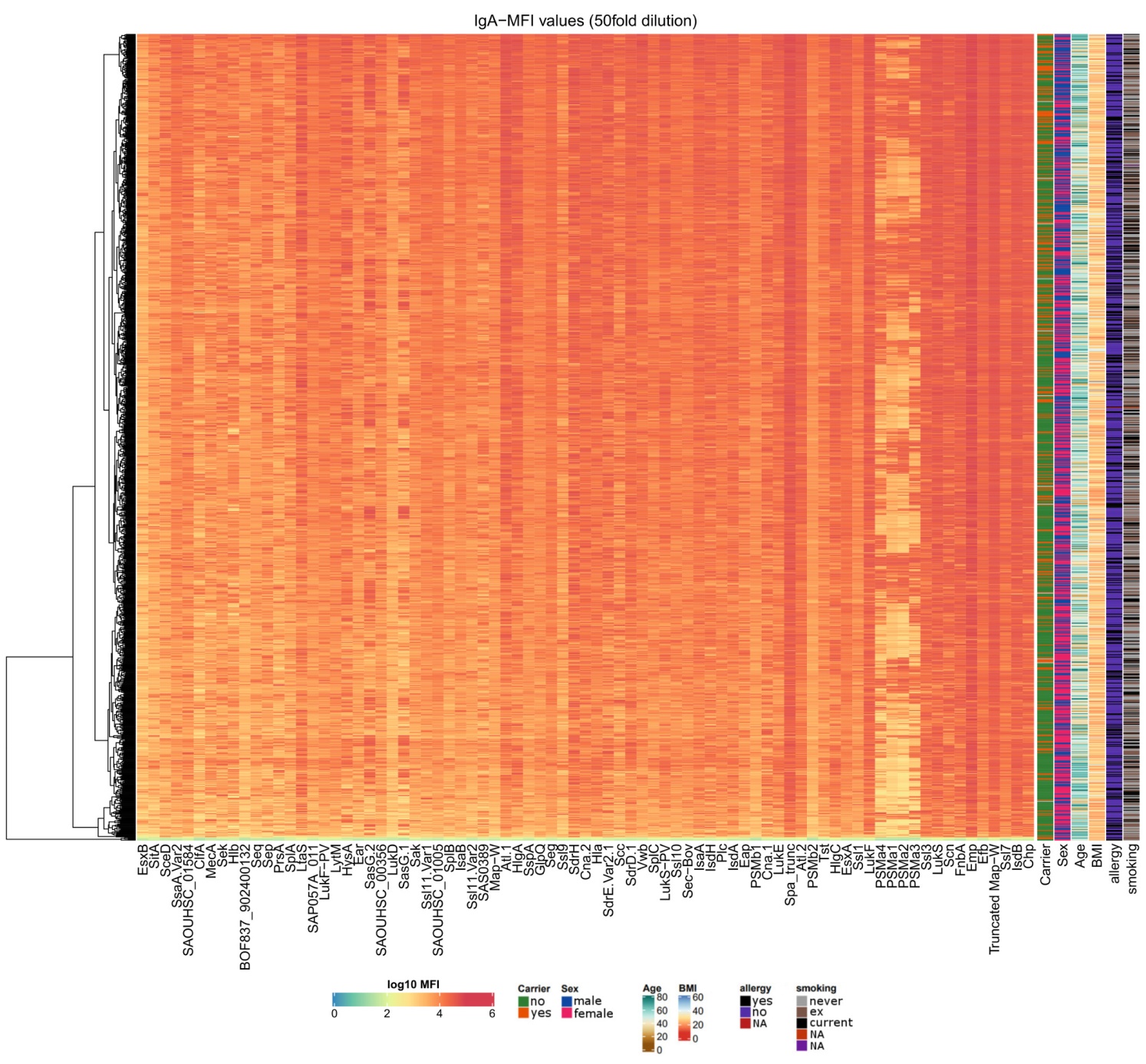


**Supplementary Figure 9.** The median fluorescense intensities in the IgA measurement of the 50-fold dilution of all 996 individuals (y-axis) were plotted against the 79 antigens (x-axis) on a log10-transformed scale. Phenotypic information on the individuals was included in the additional columns on the right of the heatmap including carriage status, sex, age, BMI, allergy, and smoking.


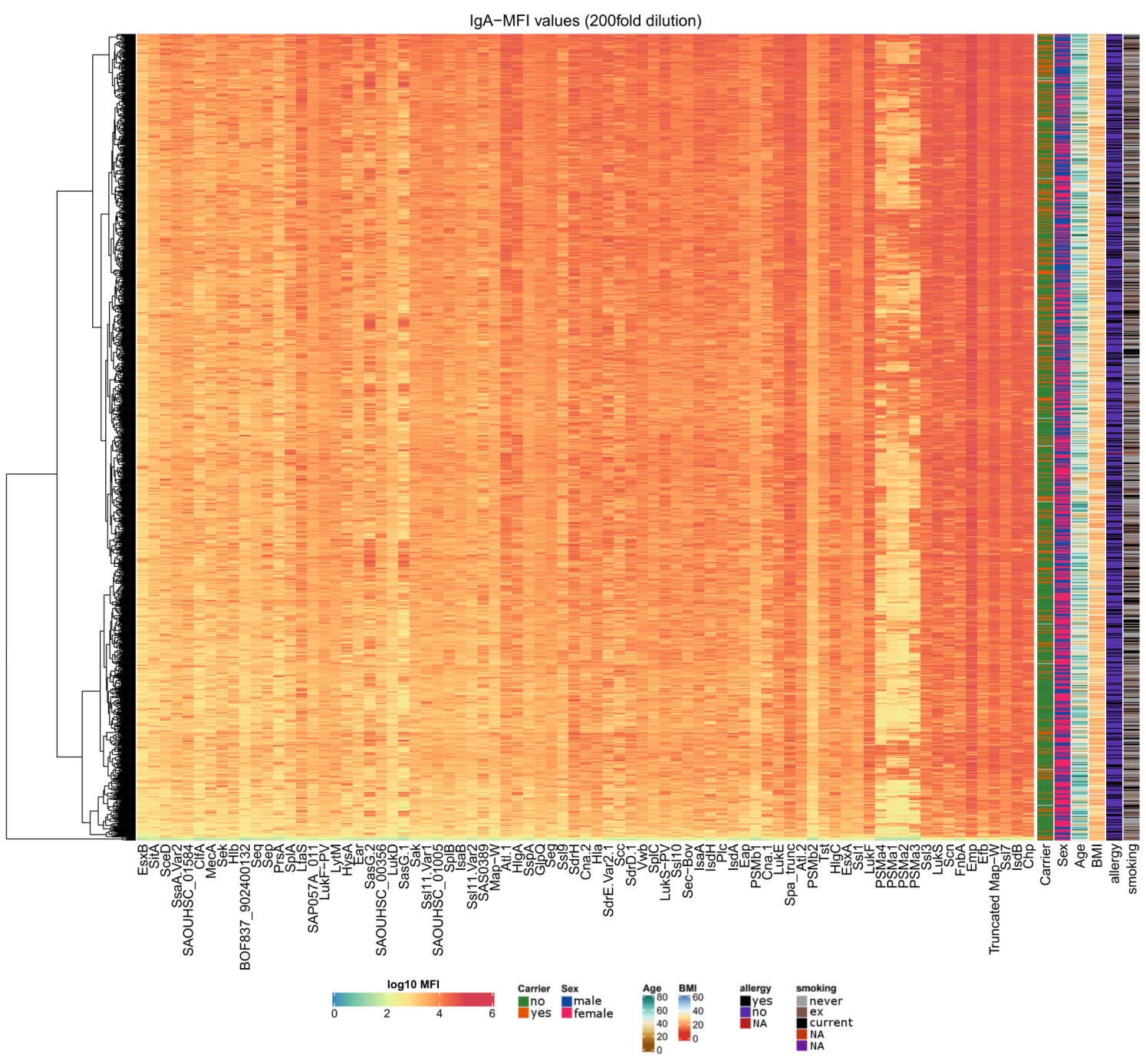


**Supplementary Figure 10.** The median fluorescense intensities in the IgA measurement of the 200-fold dilution of all 996 individuals (y-axis) were plotted against the 79 antigens (x-axis) on a log10-transformed scale. Phenotypic information on the individuals was included in the additional columns on the right of the heatmap including carriage status, sex, age, BMI, allergy, and smoking.


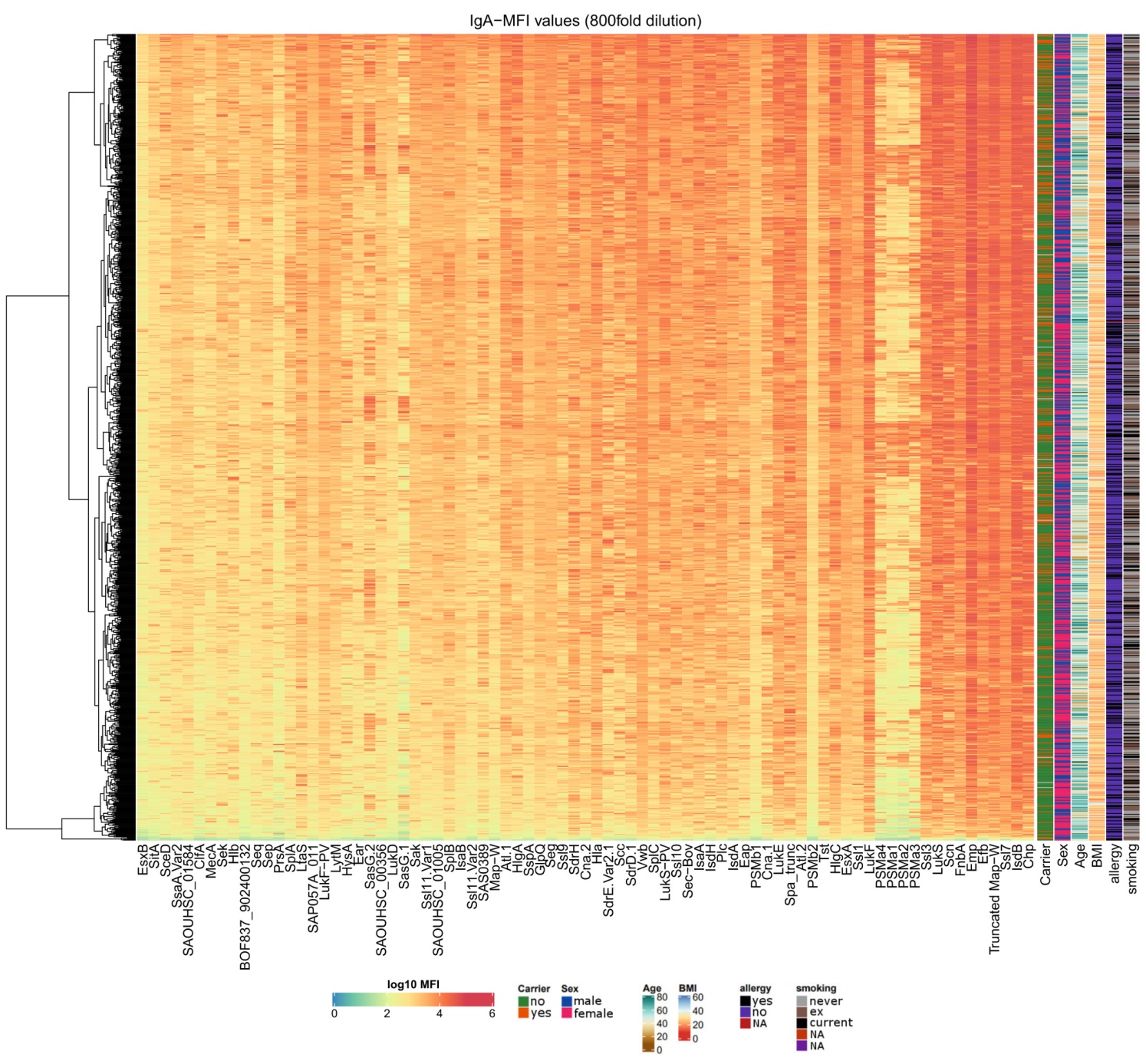


**Supplementary Figure 11.** The median fluorescense intensities in the IgA measurement of the 800-fold dilution of all 996 individuals (y-axis) were plotted against the 79 antigens (x-axis) on a log10-transformed scale. Phenotypic information on the individuals was included in the additional columns on the right of the heatmap including carriage status, sex, age, BMI, allergy, and smoking.


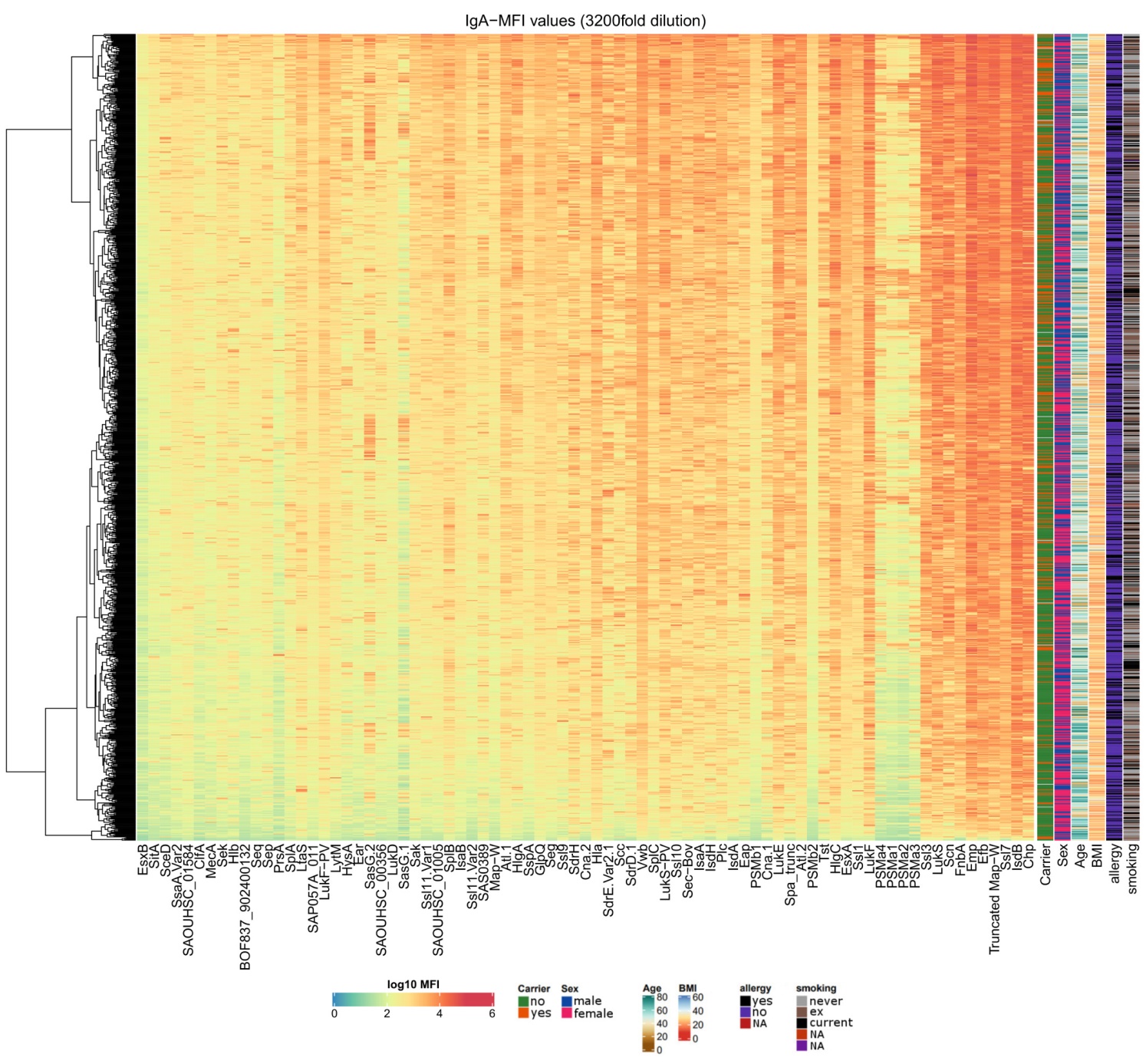


**Supplementary Figure 12.** The median fluorescense intensities in the IgA measurement of the 3,200-fold dilution of all 996 individuals (y-axis) were plotted against the 79 antigens (x-axis) on a log10-transformed scale. Phenotypic information on the individuals was included in the additional columns on the right of the heatmap including carriage status, sex, age, BMI, allergy, and smoking.


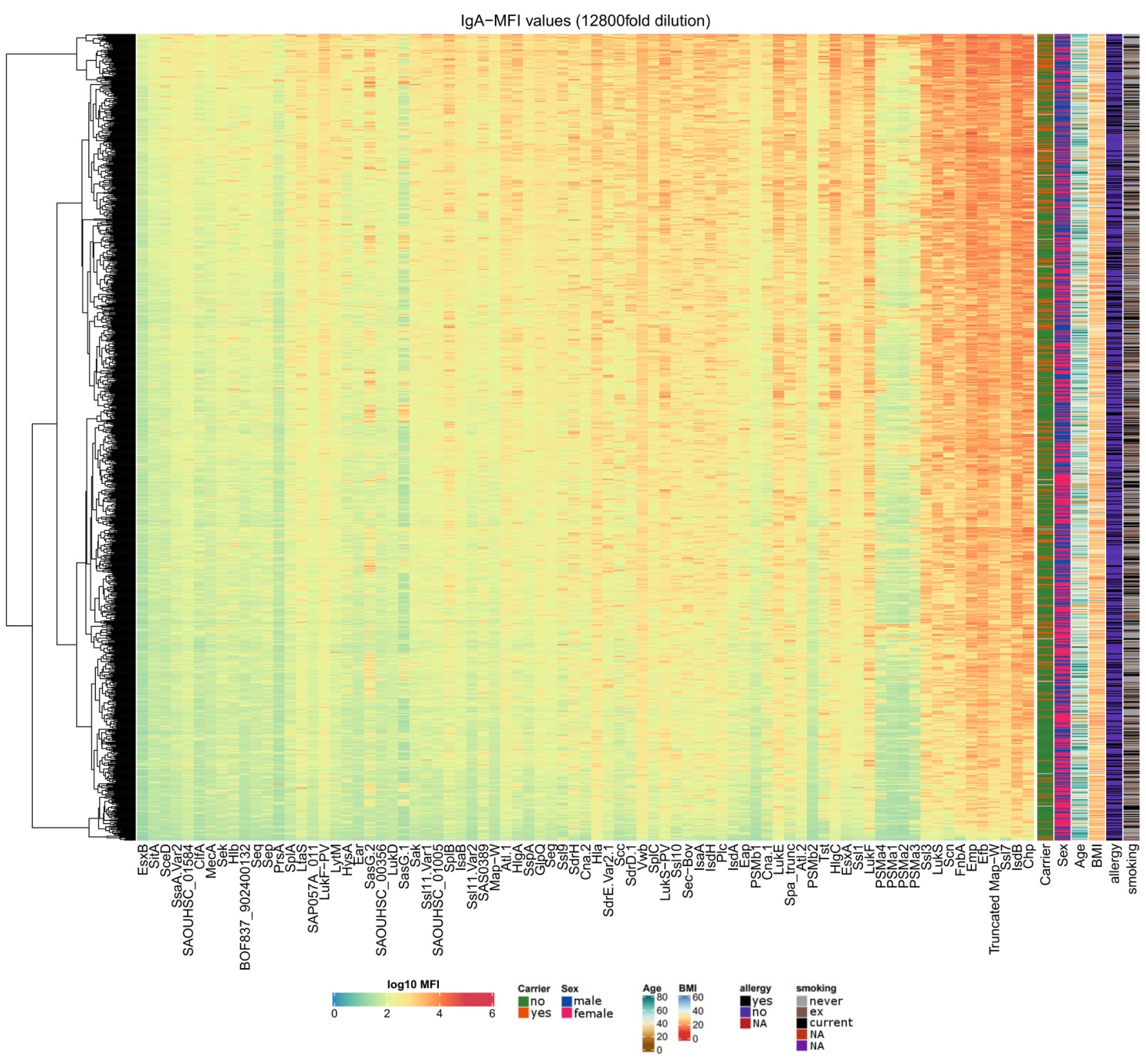


**Supplementary Figure 13.** The median fluorescense intensities in the IgA measurement of the 12,800-fold dilution of all 996 individuals (y-axis) were plotted against the 79 antigens (x-axis) on a log10-transformed scale. Phenotypic information on the individuals was included in the additional columns on the right of the heatmap including carriage status, sex, age, BMI, allergy, and smoking.


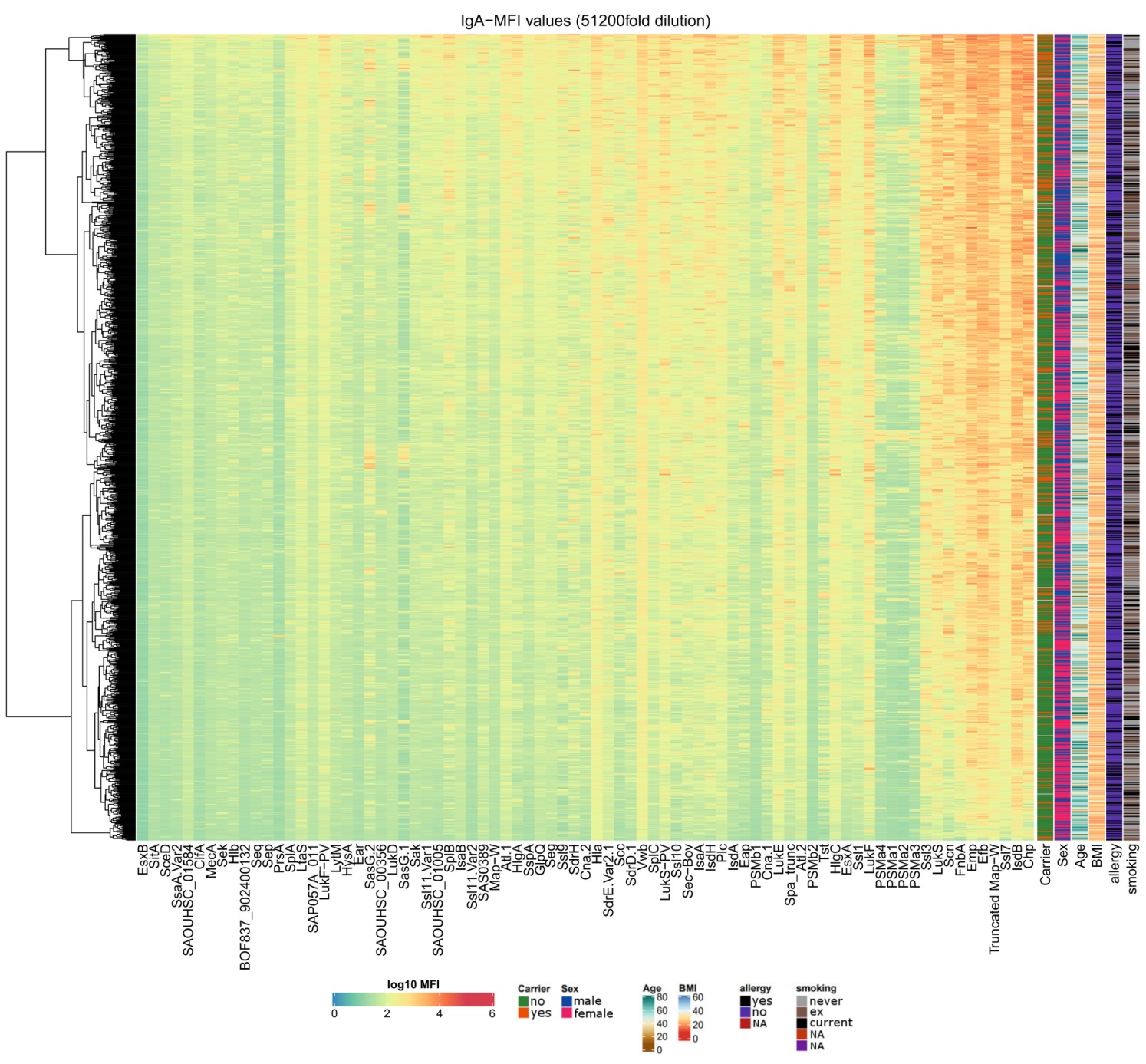


**Supplementary Figure 14.** The median fluorescense intensities in the IgA measurement of the 51,200-fold dilution of all 996 individuals (y-axis) were plotted against the 79 antigens (x-axis) on a log10-transformed scale. Phenotypic information on the individuals was included in the additional columns on the right of the heatmap including carriage status, sex, age, BMI, allergy, and smoking.


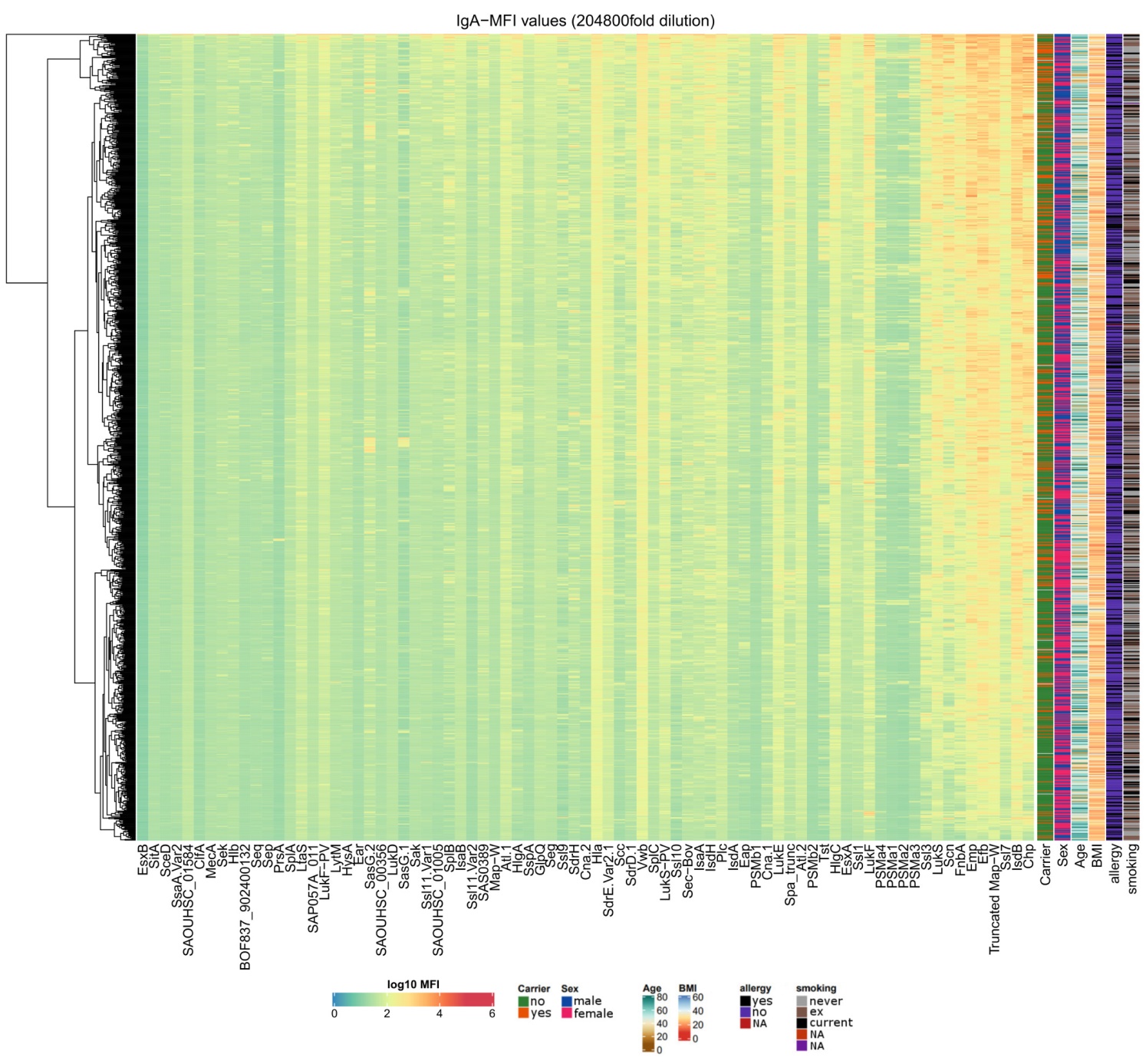


**Supplementary Figure 15.** The median fluorescense intensities in the IgA measurement of the 204,800-fold dilution of all 996 individuals (y-axis) were plotted against the 79 antigens (x-axis) on a log10-transformed scale. Phenotypic information on the individuals was included in the additional columns on the right of the heatmap including carriage status, sex, age, BMI, allergy, and smoking.


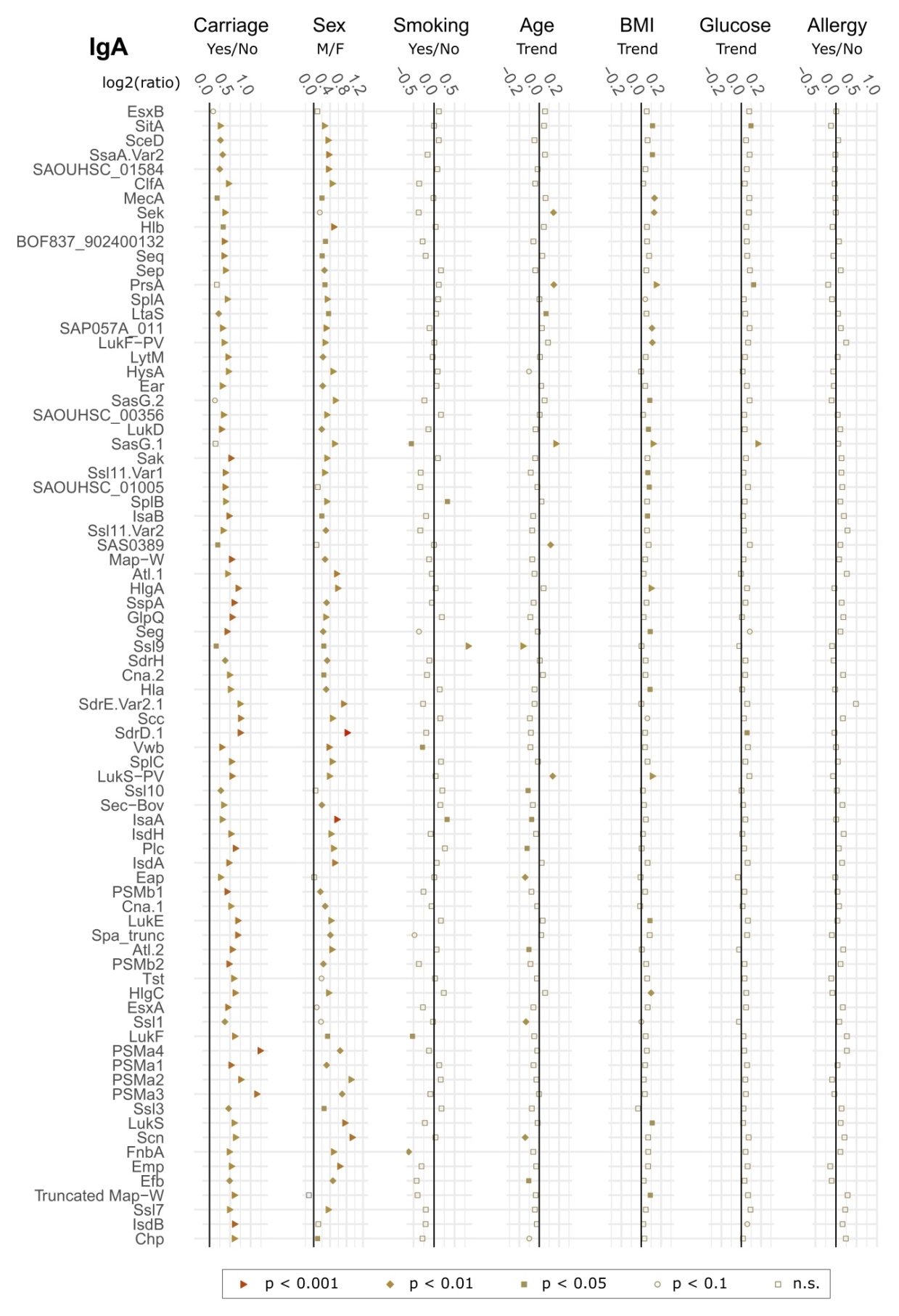


**Supplementary Figure 16.** Antigen-wise depiction of IgA derived ratios from group comparisons. The log2 ratio is shown on the x-axis in each panel for all 79 antigens on the y-axis. The significance value of each comparison is encoded in color and shape as shown in the bottom legend.


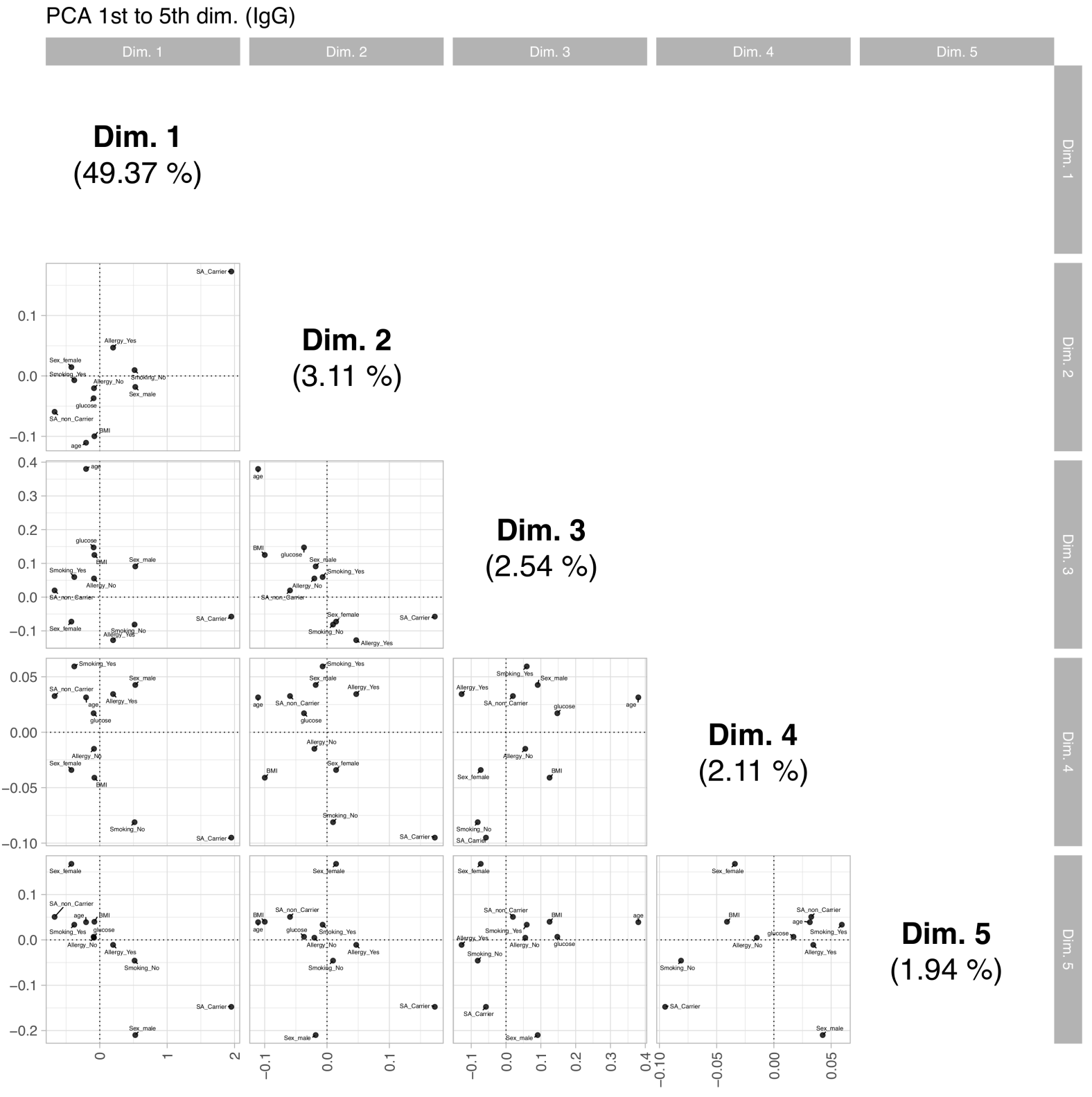


**Supplementary Figure 17.** Principal component analysis showing the influence of phenotypes for the anti-staphylococcal IgG repertoire. The plots decipt the first until the fifth dimension against each other as indicated on the upper and right border.


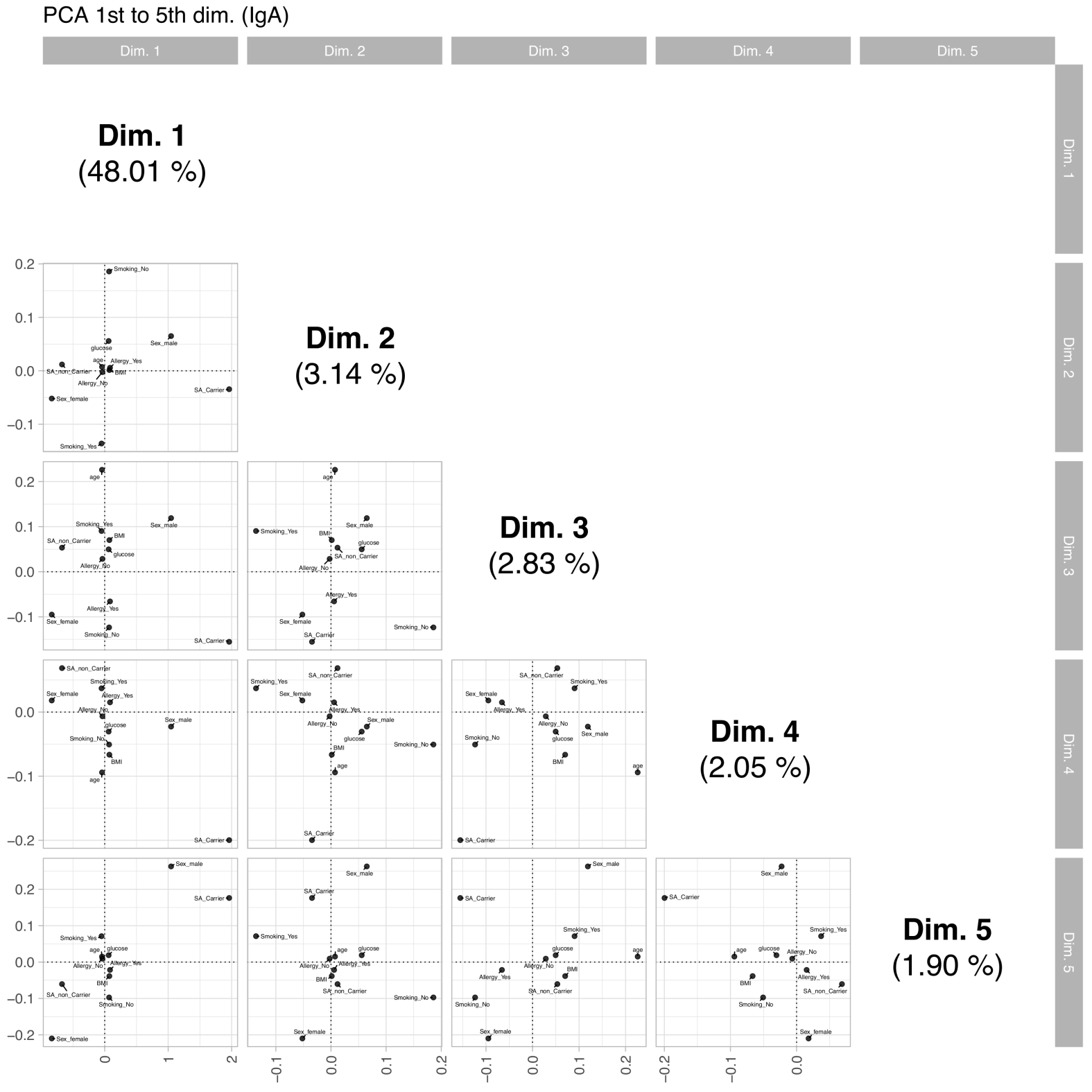


**Supplementary Figure 18.** Principal component analysis showing the influence of phenotypes for the anti-staphylococcal IgA repertoire. The plots decipt the first until the fifth dimension against each other as indicated on the upper and right border.


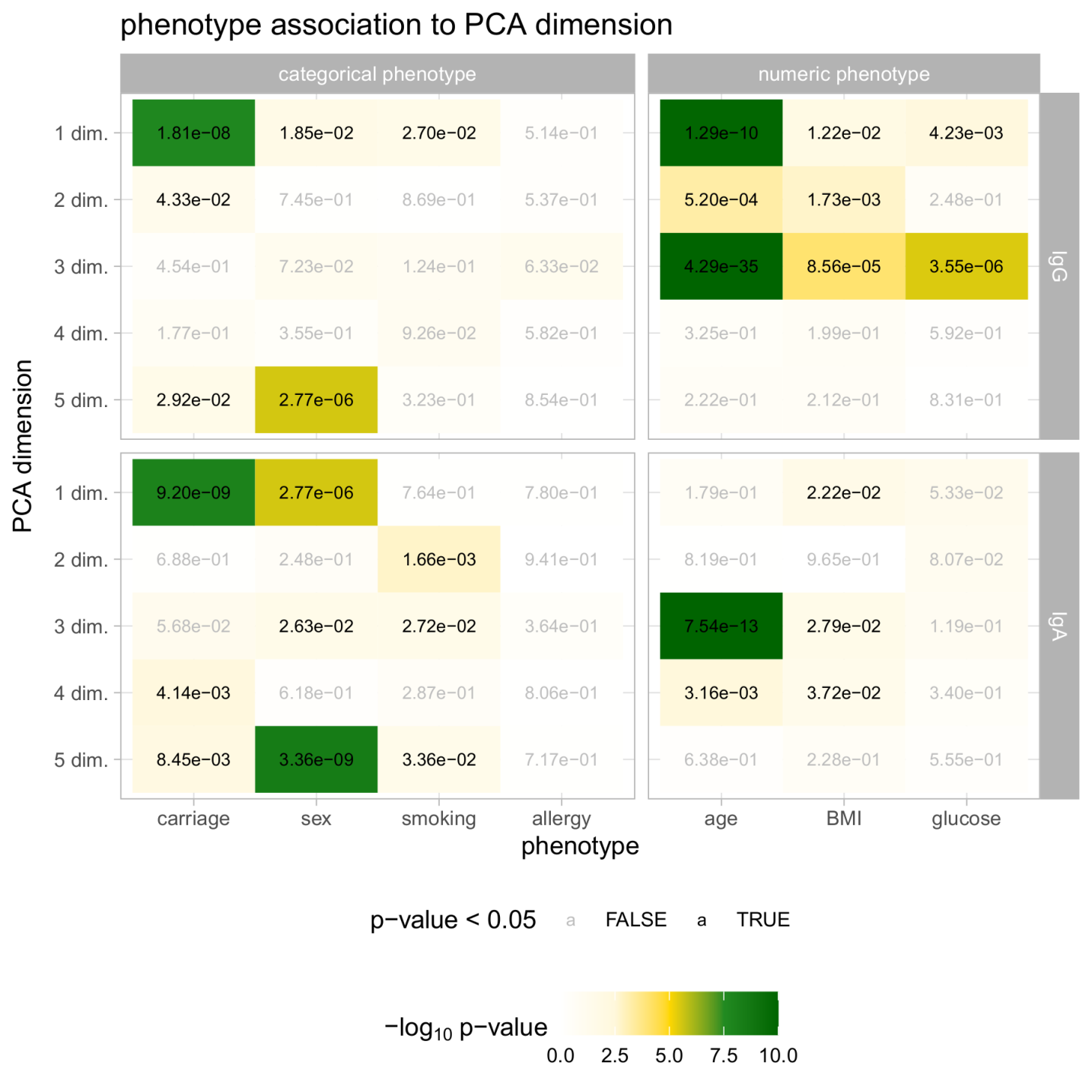

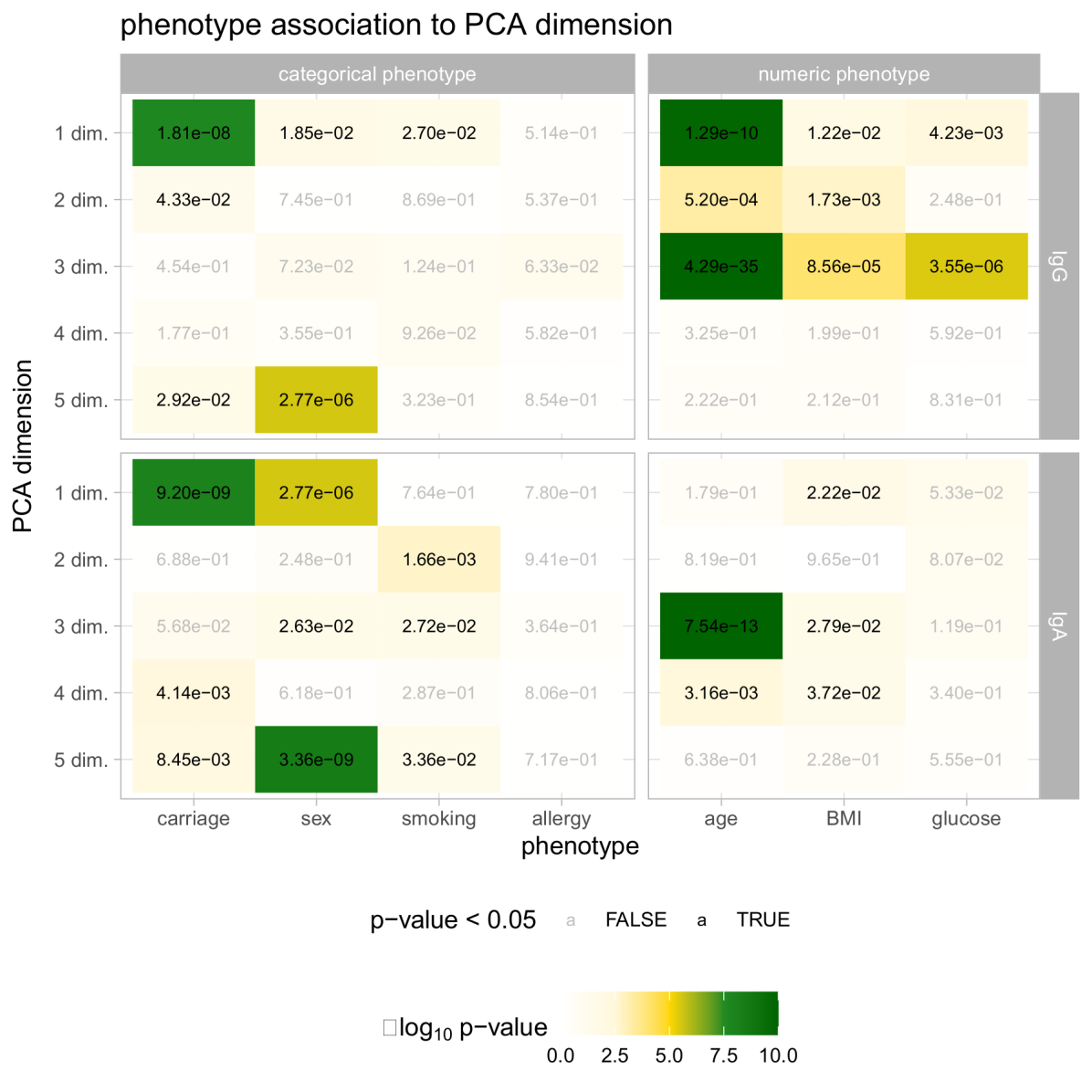


**Supplementary Figure 19.** Association results of all included phenotypes with each of the first five principal components across two antibody species (IgG in upper panel, IgA in lower panel). The association for each phenotype and PCA dimension combination per antibody species was determined by either binomial generalized linear models with logit link function for categoric phenotypes (carriage, sex, smoking, allergy in left panel) or standard Gaussian generalized linear models for numeric phenotypes (age, BMI, serum glucose in right panel).


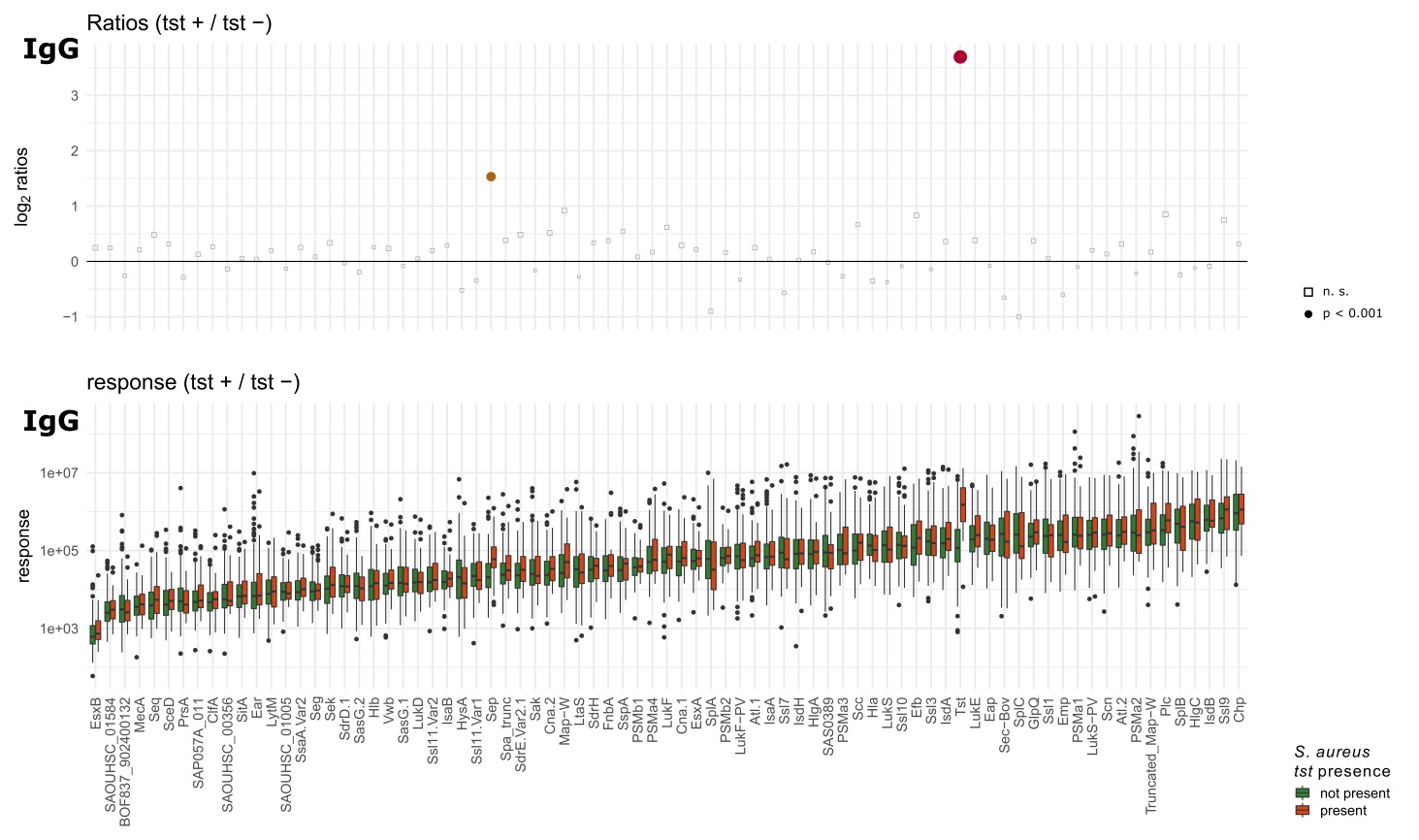


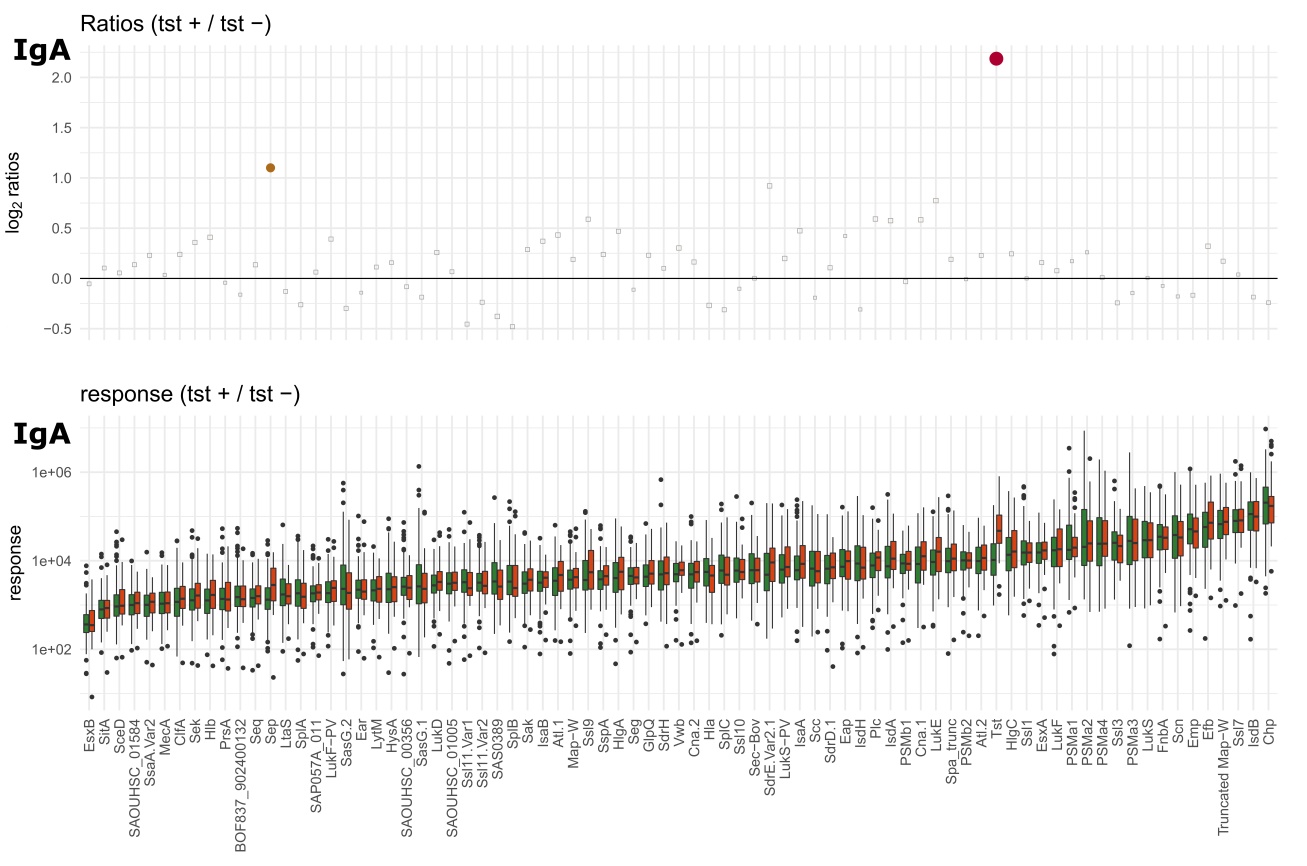


**Supplementary Figure 20.** Comparison of antibody responses in *S. aureus* carriers colonized with strains carrying *tst* to carriers colonized with strains lacking *tst*.
